# The RosR transcription factor is required for gene expression dynamics in response to extreme oxidative stress in a hypersaline-adapted archaeon

**DOI:** 10.1186/1471-2164-13-351

**Published:** 2012-07-30

**Authors:** Kriti Sharma, Nicholas Gillum, J Lomax Boyd, Amy Schmid

**Affiliations:** 1 Center for Systems Biology, Institute for Genome Sciences and Policy, Durham, NC, 27710, USA; 2 Biology Department, Duke University, Durham, NC, 27710, USA

**Keywords:** *Halobacterium salinarum*, Oxidative stress, Gene regulation, Transcription factor, Archaea

## Abstract

**Background:**

Previous work has shown that the hypersaline-adapted archaeon, *Halobacterium salinarum NRC-1*, is highly resistant to oxidative stress caused by exposure to hydrogen peroxide, UV, and gamma radiation. Dynamic alteration of the gene regulatory network (GRN) has been implicated in such resistance. However, the molecular functions of transcription regulatory proteins involved in this response remain unknown.

**Results:**

Here we have reanalyzed several existing GRN and systems biology datasets for *H. salinarum* to identify and characterize a novel winged helix-turn-helix transcription factor, VNG0258H, as a regulator required for reactive oxygen species resistance in this organism. This protein appears to be unique to the haloarchaea at the primary sequence level. High throughput quantitative growth assays in a deletion mutant strain implicate VNG0258H in extreme oxidative stress resistance. According to time course gene expression analyses, this transcription factor is required for the appropriate dynamic response of nearly 300 genes to reactive oxygen species damage from paraquat and hydrogen peroxide. These genes are predicted to function in repair of oxidative damage to proteins and DNA. *In vivo* DNA binding assays demonstrate that VNG0258H binds DNA to mediate gene regulation.

**Conclusions:**

Together these results suggest that VNG0258H is a novel archaeal transcription factor that regulates gene expression to enable adaptation to the extremely oxidative, hypersaline niche of *H. salinarum*. We have therefore renamed VNG0258H as RosR, for *r*eactive *o*xygen *s*pecies *r*egulator.

## Background

*Halobacterium salinarum,* an extremely halophilic euryarchaeon that resides in salt lakes and marine salterns, requires nearly saturated salt for growth and survival (100–150 g/L) [1]. In these environments, UV damage from intense sunlight and desiccation-rehydration cycles generate high levels of reactive oxygen species (ROS) and damage DNA and proteins [2]. *H. salinarum* is highly resistant to ROS damage, withstanding many times what *E. coli* and other radiation-sensitive organisms can survive [3]. Like other ROS-resistant microbes such as *Deinococcus radioduransH. salinarum* uses a battery of enzymatic and non-enzymatic strategies to withstand macromolecular damage. These include functional redundancy of DNA repair and antioxidant enzyme-coding genes [4-6]; a high cytosolic Mn(II) to Fe(III) ratio [7-9]; genomic polyploidy to provide templates for DNA double strand break repair [10]; and differential regulation of genes encoding macromolecular repair functions in response to oxidative stress [11].

Particularly striking is the effect of ROS on the gene regulatory network (GRN) of *H. salinarum.* Computational inference methods on global gene expression data suggest that more than 80 predicted DNA binding proteins work together to bring about a concerted, dynamic gene expression response to neutralize ROS toxicity and repair macromolecular damage [11]. In addition, the molecular functions of these putative ROS-responsive regulators remain unclear in this organism and other archaeal species*.*

Transcription mechanisms in archaea are a chimera of eukaryotic and bacterial components. General transcription factors in archaea (e.g. TATA-binding protein and TFIIB homologs) more closely resemble those of eukaryotes, whereas archaeal activators and repressors resemble those of bacteria [12]. Bacterial-type transcription factors (TFs) of the helix-turn-helix class of DNA binding proteins are particularly overrepresented in available sequenced archaeal genomes [13-15]. Compared with the substantial information on TF function in other domains of life, relatively few of the ~4,000 predicted archaeal TFs [14] have been assigned a known function *in vivo* despite intense interest in recent years [16-25].

Here we identify and characterize the function of VNG0258H, a putative TF comprised of a winged helix-turn-helix (wHTH) domain and an uncharacterized domain unique to a subset of haloarchaeal species. We used existing systems biology datasets to generate the hypothesis that VNG0258H may function in the response to ROS and/or oxygen perturbations. To test this, we generated a *VNG0258H* deletion mutant and monitored global gene expression dynamics and high throughput growth physiology in this strain in response to hydrogen peroxide (H_2_O_2_) and paraquat (PQ). Results suggest that VNG0258H is required for ROS resistance and modulates the expression of genes encoding proteins involved in repairing cellular damage from extremely high levels of reactive oxygen species (ROS). *In vivo* binding assays demonstrate that VNG0258H binds directly to the promoter of *sod2*, encoding the [Mn] superoxide dismutase. We conclude that VNG0258H is a unique haloarchaeal TF required for the response to extreme oxidative stress endemic to hypersaline environments. We have therefore renamed VNG0258H as RosR, *r*eactive *o*xygen *s*pecies *r*egulator.

## Methods

### Strains and growth conditions

All strains used in this study are listed in Additional file 1: Table S4. Briefly, *Halobacterium salinarum* NRC-1 (ATCC700922) was used to determine the *in vivo* function of VNG0258H. A strain harboring an in-frame deletion of *VNG0258H* was constructed in the *Δura3* uracil auxotroph parent strain as described previously [26]. *H. salinarum* strains harboring *VNG0258H* fused to the c-myc epitope at its C-terminus and driven by the *VNG2293G* strong constitutive promoter on a low-copy number plasmid was constructed as described previously [27]. For culturing the strains carrying the *VNG0258H::c-myc* and *trmB::c-myc* fusions (used for ChIP-qPCR and growth assays), cultures were supplemented with 20 μg/mL mevinolin for plasmid maintenance. For routine culturing, *H. salinarum Δura3* parent and *Δura3ΔVNG0258H* deletion mutant strains were grown in complete medium (CM; 250 g/L NaCl, 20 g/L MgSO_4_·7H_2_O, 3 g/L sodium citrate, 2 g/L KCl, 10 g/L peptone) supplemented with uracil (50 mM) to complement the *Δura3* auxotrophy.

### High throughput growth curves

Starter cultures of *H. salinarum* NRC1, *Δura3* parent strain, Δ*VNG0258H*, or Δ*VNG0258H* cells complemented with *VNG0258::c-myc* on a plasmid (Additional file 1: Table S4) were grown to OD600 ~1.0 in 50 mL CM supplemented with 50 mM uracil. Culture aliquots (200 μL) were grown at 37°C for 48 hours under continuous shaking (~225 rpm) in a Bioscreen C microbial growth analyzer (Growth Curves USA, Piscataway, NJ) set to measure optical density at 600 nm automatically every 30 minutes for 200 culture samples simultaneously. For continuous H_2_O_2_ and paraquat (PQ) exposure experiments, cultures were diluted in CM-uracil to OD600 ~0.05 and supplemented with 30% (v/v) H_2_O_2_ to final concentrations of 5, 6, 7, 12.5, 18.75, or 25 mM H_2_O_2_. 100 mM PQ was added to final concentrations of 0.083, 0.167, or 0.333 mM. These ROS conditions have been used previously and are also used here as proxies for the continuous high-level UV exposure that *H. salinarum* experiences on a routine basis in its salt lake habitat [11,28]. For shock experiments, oxidant was added to growing cultures in logarithmic phase at OD_600_ 0.250 to 0.375 (as measured in a standard 1x1 cm path-length cuvette spectrophotometer). At least 4 biological replicate trials were conducted for each strain under each condition. Growth rate was calculated independently for each growth curve by taking the slope of the linear regression fit to log_2_-transformed curves from 12 to 24 hours for continuous exposure experiments, and from 20 to 32 hours for shock experiments. Individual growth rates were then averaged by strain and growth condition. Averages, standard deviations, and results of non-parametric paired *t*-tests (comparing *H. salinarum Δura3* to *ΔVNG0258H* strain growth under each condition) are reported in the Figures. See Additional file 2 for supplementary methods regarding growth assays. See Additional file 3: Table S1 for all raw and analyzed growth data.

### Gene expression microarray sample preparation, hybridization, and data analysis

*H. salinarum Δura3* parent and Δ*VNG0258H* mutant strains were grown in CM supplemented with uracil to mid-logarithmic phase (OD_600_ ~ 0.5). For H_2_O_2_ time courses, 4-mL culture aliquots were removed for RNA extraction at three time points prior to the addition of 25 mM H_2_O_2_ (−40 min, -20 min, 0 min) and five time points following H_2_O_2_ addition (10, 20, 40, 60, 80 minutes). Paraquat (PQ) time courses were prepared similarly with the exception that additional time points were taken at 2 h, 8 h, and 24 h after the addition of 0.333 mM PQ to assess long-term expression patterns. For each biological duplicate time course, all samples were removed from the same culture to ensure coherence of gene expression between unstressed and stressed cultures. From each sample, cells were immediately pelleted by centrifugation (12,000 *g*, 30 sec, 25°C) and snap-frozen in liquid nitrogen. Sample pellets were stored overnight at −80°C, followed by RNA preparation using the Absolutely-RNA kit (Stratagene, La Jolla, CA) according to the manufacturer’s instructions. RNA quality was assessed using the Bioanalyzer 2100 (Agilent Technologies, Santa Clara, CA). Freedom from DNA contamination was ensured by PCR amplification of 200 ng of each RNA sample. 600 ng of each quality-checked RNA sample directly labeled with Cy3 and Cy5 dyes (Kreatech) as described previously [29,30] and combined in equimolar amounts with oppositely labeled *H. salinarum* NRC-1 reference RNA (from batch cultures grown in CM at 37°C to mid-logarithmic phase). This common reference RNA was used across all ~950 microarray experiments listed in the *H. salinarum* NRC-1 microarray data repository [31]. Samples were hybridized to a custom 60-mer oligonucleotide microarray (Agilent technologies, Santa Clara, CA, 8 x 15,000 feature array, AMADID ID #30108, GEO platform accession GPL14876). This array contains 2,410 non-redundant open reading frames (ORFs) of the *H. salinarum* NRC-1 genome. Probes for each ORF were spotted on each array six-fold and dye-swapping was conducted (to rule out bias in dye incorporation) for all samples, yielding 12 technical replicates per gene per time point. Slide hybridization and washing protocols were performed according to the manufacturer’s instructions, except that hybridization was conducted in the presence of 37.5% formamide at 68°C to ensure proper stringency due to the high G + C content of the *H. salinarum* genome (67%, [32]).

Slide scanning and spotfinding were conducted using Feature Extraction software (Agilent). Within the R Bioconductor [33] m-array and limma packages [34], resultant raw data were background-subtracted using normexp [35], Loess normalized within each array, and quantile normalized between all arrays. Any of the 12 gene-specific probes for each gene lying outside the 99^th^% confidence interval were removed using Dixon’s test [36]. Finally, remaining probe intensities for each gene were averaged and log_2_ ratios were calculated, yielding one expression ratio per gene. Resultant processed data are listed in Additional file 4: Table S2 and Additional file 5: Table S3. Both raw and processed microarray data are also available through the NCBI Gene Expression Omnibus (GEO) accession number GSE33980.

### *In vivo* DNA binding assays with ChIP-qPCR

*H. salinarum* harboring *VNG0258H::myc* was grown to mid-logarithmic phase (OD_600_ ~ 0.5) in CM supplemented with mevinolin. Transcription factor-chromatin complexes were then cross-linked *in vivo* with 1% formaldehyde for 20 min at room temperature and subjected to immunoprecipitation (IP) by virtue of the myc epitope tag as described previously [27]. Primers (Integrated DNA Technologies, Coralville, IA) were designed according to criteria described in [37] and are listed in Additional file 1: Table S4. ChIP samples from *trmB::c-myc* cells were run simultaneously as controls, since TrmB is a transcription factor previously shown not to bind the region of interest [20]. Quantitative PCR (qPCR) reaction and thermocycling conditions were as described in [27]. Each of the five biological replicate samples of RosR ChIP were run in triplicate qPCR reactions for a total of 15 data points per sample. Reactions with C_T_ values greater than 0.5 standard deviations from the triplicate mean were excluded from analysis. Enrichment of RosR binding at each promoter locus was calculated in each ChIP sample compared to the input sample using relative quantitation as described [27]. Resultant data reported represent the mean of all trials ± SEM.

### Systems biology data analysis, integration, and visualization

All systems biology datasets were analyzed and visualized in the context of the web executable, interoperable Gaggle data analysis environment [38] and other existing online database tools. Specifically, predictions and hypotheses were made using the existing GRN for *H. salinarum*[11] and explored in Cytoscape [39]. Amino acid sequences of VNG0258H homologs from other halophilic archaea were compared using PSI-BLAST [40] in the context of the Halolex database [41] and NCBI GeneBank. Sequences were aligned using ClustalW [42]. Transcriptome structure data for the VNG0258H genomic locus [43] (see Results) were visualized using the *H. salinarum* genome database [44] and the Gaggle Genome Browser [45].

The TM4 MultiExperiment Viewer (MeV) application [46] within the Gaggle environment was used for statistical analysis of microarray gene expression datasets. Specifically, Significance Analysis of Microarrays (SAM, a *t*-test-based method) was used to detect gene groups with significantly different expression levels in the *Δura3* parent and *ΔVNG0258H* mutant strains. Genes significantly up- or down-regulated in *ΔVNG0258H* were considered to be VNG0258H-dependent. Genes with significantly different expression in PQ or H_2_O_2_*vs.* standard conditions in the Δ*ura3* parent strain but not Δ*VNG0258H* were considered to be PQ or H_2_O_2_-responsive but VNG0258H-independent. The latter group was further subjected to KMEANS analysis to detect genes with altered dynamics in Δ*VNG0258H* cells. Annotations for genes within resultant clusters were analyzed using the Firegoose web portal within the Gaggle [47]. Annotated genes were subsequently grouped by arCOG annotations [48] using the R Bioconductor package within the Gaggle environment. Significance of enrichment within arCOG categories was calculated using term-for-term analysis as described [49].

*Cis*-regulatory sequence predictions were conducted using the MEME online software package [50] with two different sequence inputs: (a) open reading frames and 500 bp upstream sequence of the 50 genes differentially expressed in both H_2_O_2_ and PQ datasets; (b) promoter sequences of the *sod2* gene from the 8 haloarchaeal genomes containing RosR homologs. Searches on each type of sequence input were constrained to 6–20 bp motifs. Palindromic output was not enforced. MEME was run in discriminative mode using the first 250 kbp of the *H. salinarum* genome as negative sequence. Output sequence position weight matrices were visualized in sequence logo format using the WebLogo package (http://weblogo.berkeley.edu).

## Results

### Using existing systems biology datasets to identify candidate regulators of reactive oxygen species (ROS) stress response

To identify transcription regulatory proteins involved in the response to ROS and/or oxygen-related physiology in *H. salinarum*, we mined the existing systems biology datasets for this organism to identify candidate transcription factors. These data types include (1) the computationally inferred GRN; (2) changes in mRNA abundance microarray data during perturbations in oxygen and ROS conditions [11,30]; (3) genome-wide transcriptome structure [43]; and (4) proteomics data [51].

**Figure 1:**
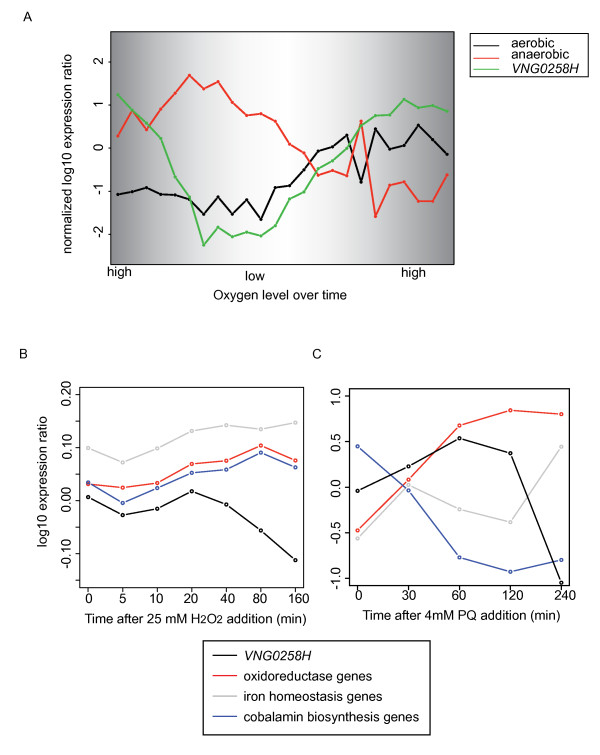
***VNG0258H*gene expression in response to H**_**2**_**O**_**2**_**and oxygen.** (**A**) Comparison of *VNG0258H* gene expression to that of genes involved in aerobic and anaerobic physiology [30]. The x-axis represents shifts in oxygen levels over time in a fermentor. Graph background shading corresponds to the relative oxygen concentration. “High” oxygen represents 100% oxygen saturation in CM medium (5 μM) as measured by a dissolved oxygen probe. “Low” represents 5% saturation or below [30]. The y-axis represents mean and variance normalized log_10_ expression ratios compared to mid-logarithmic phase *H. salinarum*. The green trace represents *VNG0258H* gene expression, whereas black and red traces represent the mean expression profiles for genes encoding proteins involved in aerobic and anaerobic physiology, respectively [30]. (**B**) Mean gene expression profiles for clusters of genes correlated with *VNG0258H* mRNA changes in response to H_2_O_2_[11]. See legend for colors. (**C**) Mean gene expression profiles in response to PQ for genes from (A). Colors are as in (B).

The existing GRN models have implicated approximately 80 TFs in the response to ROS [11,31]. However, some of these TFs exhibit similar changes in mRNA abundance in response to ROS, and so the inference procedure frequently groups several TFs into a single regulatory node [31]. Thus, the computational inference procedure cannot discern which TF within a group regulates which target genes, nor can it distinguish direct from indirect regulatory influences. We reasoned that slight differences in the expression profiles of TFs within the same node may not have been detected by the inference procedure, but may become evident upon closer inspection. We therefore re-examined the gene expression profiles of each of the 80 TFs in the GRN under oxidative conditions (i.e. in the presence of high oxygen, paraquat, or hydrogen peroxide). In response to changes in oxygen levels, the expression pattern of one putative TF, *VNG0258H,* ranks first of all the TFs in the genome in (1) correlation with genes associated with aerobic physiology, including TCA cycle and electron transport (C_P_ = 0.327; Figure 1A) [30]; (2) anti-correlation with genes associated with anaerobic physiology, including DMSO reduction and phototrophy (C_P_ = −0.621; Figure 1A) [30]; and (3) magnitude of change (3.5-fold down-regulated with low oxygen and 2-fold up-regulated in high oxygen). In response to hydrogen peroxide (H_2_O_2_) exposure [9,11], *VNG0258H* expression is anti-correlated with clusters of genes associated with cobalamin biosynthesis, iron homeostasis, and redox reactions (Figure 1B). Gene expression correlations are more complex in response to the redox cycling drug paraquat (PQ), with some clusters correlated and others anti-correlated with the *VNG0258H* expression profile (Figure 1C). 

Genome-wide whole transcript mapping data indicates that the *VNG0258H* gene is transcribed as a monocistronic message flanked by genes of unknown function [43]. The *VNG0258H* protein product has been detected by mass spectrometric proteomics in the presence of oxygen [30] and during recovery from high levels of gamma radiation [2,51], confirming that the annotated ORF encodes a *bona fide* protein that is expressed under similar conditions as the *VNG0258H* transcript. Based on this new perspective on existing systems biology datasets for *H. salinarum*, we hypothesize that *VNG0258H* encodes a putative TF that may play a role in the response to oxygen and/or oxidative stress conditions.

### Sequence homology suggests that VNG0258H may represent a class of DNA binding proteins unique to haloarchaea

**Figure 2:**
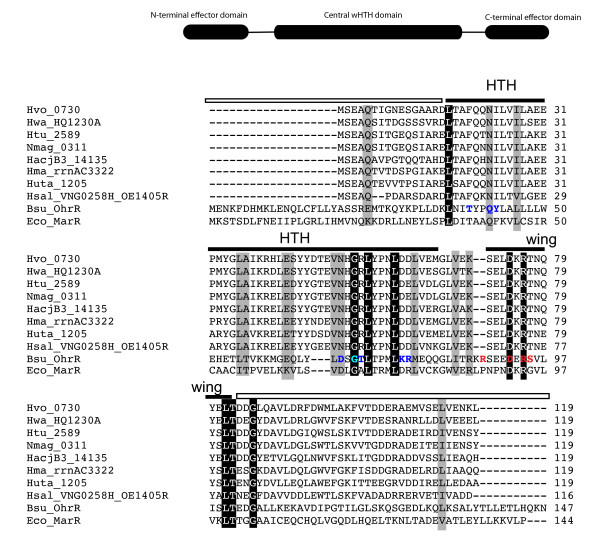
**Homology of VNG0258H winged helix-turn-helix (wHTH) putative transcription factor with haloarchaeal homologs and bacterial matches to wHTH domain.** Residues in bold blue font depict those known to interact with the major groove of OhrR in *B. subtilis*, whereas those in bold red letters represent residues of the wing that contact the minor groove [52]. In the N- or C-terminal domains (white overbar), no homology was detected outside the halophilic archaea. Perfectly conserved residues are shaded black, whereas conservatively substituted residues are shaded grey. Black overbars designate characterized helix-turn-helix (HTH) and wing regions from bacterial MarR family members. Hvo_0730, *Haloferax volcanii* (GenBank genome accession NC002945); Hwa, *Haloquadratum walsbyi* (NC_008212); Htu, *Haloterrigena turkmenica* (NC_013743); Nmag, *Natrialba magadii* (NC_013922); Huta, *Halorhabdus utahensis* (NC_013158); HacjB3, *Halalkalicoccus jeotagali.B3* (NC_014297); Hsal, *Halobacterium salinarum* NRC-1 (NC_002607); Hma, *Haloarcula marismortui* (NC_006396); Bsu, *Bacillus subtilis*; Eco, *E. coli*. Numbers following each species name refer to gene unique identifiers in each genome. OE1405R in *H. salinarum* is a cross-reference to the corresponding gene in the R1 strain [41].

Primary amino acid sequence homology suggests that VNG0258H contains a central domain that bears weak amino acid sequence homology (39% identity, E-value < 0.007) to the GntR winged helix-turn-helix (wHTH) superfamily of bacterial transcription factors, which includes the MarR and PadR families (PFAM 03551, Figure 2). Compared to characterized bacterial MarR family members, one residue in the VNG0258H HTH region known to be important for binding the major groove of DNA is conserved, as are two residues in the wing region that bind the minor groove (Figure 2, [52]). PSI-BLAST searches with the whole VNG0258H protein sequence and the short N- and C-terminal domains flanking the central wHTH domain of VNG0258H matched only those from a small clade of halophilic archaea (Figure 2). Together these sequence data are consistent with the hypothesis that VNG0258H may represent a class of transcription factors unique to the haloarchaea. To our knowledge, none of these putative archaeal DNA binding proteins has been functionally characterized. 

### *ΔVNG0258H* growth is impaired in the presence of H_2_O_2_ and paraquat

**Figure 3:**
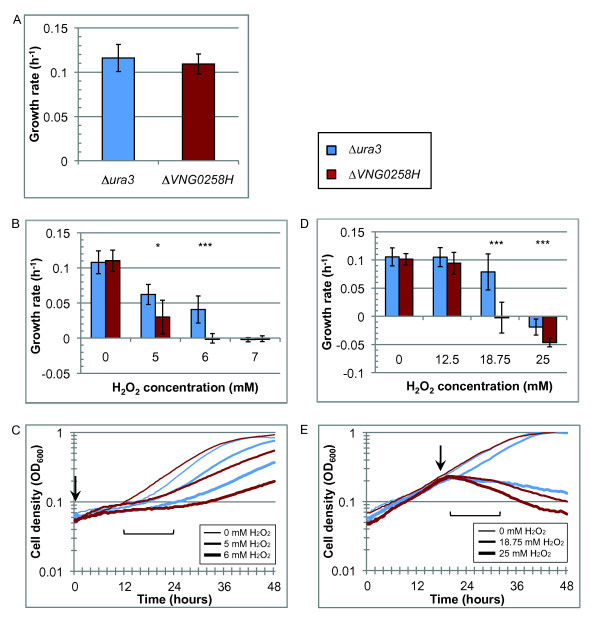
***ΔVNG0258H*is impaired for growth and survival upon exposure to hydrogen peroxide (H**_**2**_**O**_**2**_**).** (**A**) Comparison of *ΔVNG0258H* and *Δura3* parent growth rates under standard conditions across all experiments (n = 63, see also Additional file 3: Table S1). (**B**) Mean growth rates for 4 biological replicate cultures treated with H_2_O_2_ in lag phase. Blue bars represent *Δura3* cultures; red bars represent *ΔVNG0258H* cultures. Concentration of H_2_O_2_ added is indicated on the x-axis. (**C**) Representative growth curves (1 of 4 biological replicates) for cultures treated with H_2_O_2_ at beginning of growth (OD600 ≈ 0.05). Line colors are as in (B). Thin, medium, and thick lines indicate H_2_O_2_ added to a final concentration of 0, 5, or 6 mM, respectively (see legend; 7 mM curves omitted for clarity). Downward arrow indicates time of H_2_O_2_ addition. Bracket indicates period for which mean growth rates were calculated. (**D**) Mean growth rates for 7 biological replicate cultures treated with H_2_O_2_ in mid-logarithmic growth phase. (**E**) Representative growth curves for cultures treated with H_2_O_2_ in mid-logarithmic phase (OD600 ≈ 0.3). Thin, medium, and thick lines indicate H_2_O_2_ added to a final concentration of 0, 18.75, or 25 mM, respectively (curves for 6.25 and 12.5 mM conditions are omitted for clarity). Downward arrow indicates time of treatment. Bracket indicates period for which mean growth rates were calculated. In all bar graphs, error bars represent standard deviation. Asterisks represent statistically significant differences between *ΔVNG0258H* and parent strain *Δura3* under the same growth conditions, where single asterisk indicates a *p*-value < 0.01, double asterisk indicates *p* < 0.001, and triple asterisk indicates *p* < 0.0001. All raw data are given in Additional file 3: Table S1.

To test the hypothesized role of VNG0258H in the response to oxygen and/or oxidative stress conditions, we generated a strain of *H. salinarum* strain deleted for *VNG0258H* (Methods). We measured its response to varying H_2_O_2_ and paraquat (PQ) concentrations in different phases of growth. Under standard aerobic growth conditions, the *H. salinarum ΔVNG0258H* mutant strain grows similarly to the isogenic *Δura3* parent strain (Figure 3A). However, when H_2_O_2_ is added, the mutant exhibits a significant growth defect (Figures 3B, C, D, E, Additional file 6: Figure S1). The greatest difference in growth rate between the Δ*VNG0258H* and Δ*ura3* strains is observed at 6 mM H_2_O_2_ added at inoculation (Figure 3B, *p* < 7.9 x 10^-7^) and 18.75 mM H_2_O_2_ added in mid-logarithmic phase (Figure 3D, *p* < 2.4 x 10^-10^). These Δ*VNG0258H* growth defects are significantly complemented *in trans* by a constitutively expressed, plasmid-borne wild type copy of the *VNG0258H* gene (Additional file 7: Figure S2; Methods). Both strains are completely growth-inhibited when challenged with 7 mM H_2_O_2_ at time of inoculation (Figures 3B and 3C) or 25 mM H_2_O_2_ added in mid-logarithmic phase (Figures 3D, 3E, and Additional file 3: Table S1), suggesting a relationship between cell density or growth phase and H_2_O_2_ resistance. Together, these phenotypic data suggest that (a) the VNG0258H protein is important for protection against oxidative stress caused by exposure to high levels of exogenous H_2_O_2_; and (b) cell density and H_2_O_2_ resistance tend to co-vary.

**Figure 4:**
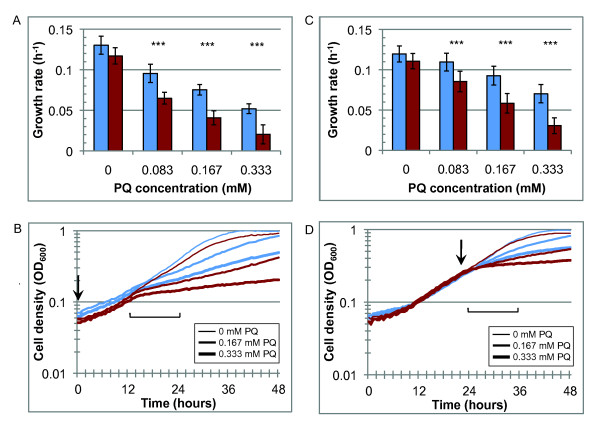
***ΔVNG0258H*is impaired for growth and survival upon exposure to paraquat (PQ).** (**A**) Mean growth rates for 7 biological replicate cultures treated in lag phase. Blue bars represent *Δura3* cultures; red bars represent *ΔVNG0258H* cultures. (**B**) Representative growth curves (1 of 7 biological replicates) for cultures treated with PQ at beginning of growth phase (OD600 ≈ 0.05). Thin, medium, and thick lines indicate PQ added to a final concentration of 0, 0.167, or 0.333 mM, respectively (curve for 0.083 mM omitted for clarity). Downward arrow indicates time of PQ addition. Bracket indicates period for which mean growth rates were calculated. Line colors are as in (A). (**C**) Mean growth rates for 7 biological replicate cultures treated with PQ in mid-logarithmic growth phase. (**D**) Representative growth curves (1 of 7 biological replicates) for cultures treated with PQ in mid-logarithmic phase (OD600 ≈ 0.3). Line widths indicate the same PQ concentrations as in (B). Representative curves for 0.083 mM condition are omitted for clarity. Asterisks and error bars are as in Figure 3. All raw data are given in Additional file 3: Table S1.

*ΔVNG0258H* is also markedly more susceptible to PQ stress than the parent strain. PQ added at the time of inoculation slows the growth rate of both strains, though *ΔVNG0258H* growth decreases more dramatically (Figure 4A; *p* < 6.0 x 10^-13^). Both strains grow normally in the presence of PQ up to about 12 hours, at which point growth rate slows significantly (Figure 4B). When PQ is added at mid-logarithmic phase, however, growth declines immediately after the PQ addition (Figure 4D). *ΔVNG0258H* is significantly more susceptible to PQ addition in mid-logarithmic phase than *Δura3*, with complete inhibition of growth observed at 0.333 mM PQ (Figure 4C; *p* < 1.6 x 10^-8^). In contrast to H_2_O_2_ response, PQ addition in lag and mid-logarithmic growth phases caused similar effects on growth, suggesting no relationship between cell density and susceptibility to PQ (*e.g.* compare Figure 4A to 4C). Together these PQ phenotypic data suggest that (a) VNG0258H is required for resistance to PQ exposure; and (b) PQ resistance of *H. salinarum* is independent of cell density.

### VNG0258H is required for appropriate gene expression dynamics in response to ROS induced by H_2_O_2_ and PQ

To determine whether VNG0258H plays a role in gene regulation, mRNA expression in the Δ*VNG0258H* deletion mutant and Δ*ura3* parent backgrounds was monitored using microarrays, 40 and 20 minutes prior to H_2_O_2_ and PQ treatment and at 10, 20, 40, 60, 80 minutes following H_2_O_2_ or PQ treatment (Additional file 8: Figure S3 and Additional file 9: Figure S4, respectively). Three additional time points at 2 h, 8 h, and 24 h were monitored for PQ. Expression was measured using microarrays spotted with probes against each of the *H. salinarum* NRC-1 open reading frames (ORFs; [43]; Methods).

#### Clusters of gene expression patterns in response to H_2_O_2_

**Figure 5:**
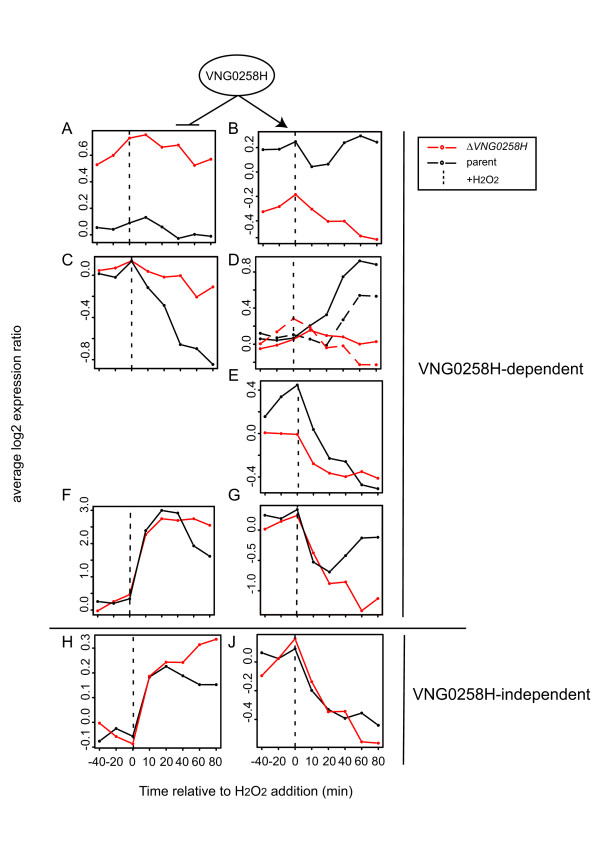
**Gene expression in response to H**_**2**_**O**_**2**_**exposure in the Δ*ura3*parent vs Δ*VNG0258H*mutant strains.** Each line in each graph represents the mean expression profile of gene clusters that rely on VNG0258H for their appropriate expression (A-G) or those that respond to 25 mM H_2_O_2_ treatment regardless of strain background (H and J). Time points before and after H_2_O_2_ exposure in the Δ*ura3* parent strain (black) or Δ*VNG0258H* mutant (red) are divided by the dotted line. (**A**) Genes requiring VNG0258H for repression regardless of condition. (**B**) Genes requiring VNG0258H for activation regardless of condition. (**C**) Genes requiring VNG0258H for repression in the presence of H_2_O_2_. (**D**) Genes requiring VNG0258H for activation in the presence of H_2_O_2_. Dotted traces represent late waves of gene expression. (**E**) Genes requiring VNG0258H for repression in the absence of H_2_O_2_. (**F**) Genes requiring VNG0258H for impulse-like dynamic induction. (**G**) Genes requiring VNG0258H for impulse-like dynamic repression. (**H**) Genes induced in response to H_2_O_2_ but independent of VNG0258H (note the difference in y-axis scale between F and H). (**J**) Genes repressed in response to H_2_O_2_ but independent of VNG0258H. Gene expression profiles for individual genes in each cluster are shown in heat maps in Additional file 8: Figure S3. Detailed annotations for genes in each cluster are listed in Additional file 4: Table S2.

As expected from previous studies [11], a substantial proportion of the genome (626 of 2,410 genes, 26%) exhibited changes in mRNA abundance in response to H_2_O_2_ treatment in the *Δura3* parent strain (Figure 5, Additional file 4: Table S2). Of these 626, 332 genes changed in abundance in response to H_2_O_2_ in the parent strain but were unaffected by the *VNG0258H* mutation (Figure 5H, J). These genes are considered “VNG0258H-independent”. 294 of the 626 genes exhibited significant changes in the Δ*VNG0258H* mutant compared to the parent during H_2_O_2_ exposure (Figure 5, Additional file 8: Figure S3). According to significance analysis of microarrays (SAM), these genes fell into four distinct patterns, or clusters, of VNG0258H-dependent induction or repression. The first cluster includes 63 genes which exhibit a change in mRNA abundance in Δ*VNG0258H* relative to Δ*ura3* regardless of H_2_O_2_ treatment (Figure 5A and B). The second cluster includes 191 genes which require VNG0258H for appropriate expression in the presence of H_2_O_2_ (Figure 5C and D). In this cluster, we detected time-resolved waves of VNG0258H-dependent activation of genes in response to H_2_O_2_ (Figure 5D, Methods), with 43 genes activated within 10 minutes of H_2_O_2_ exposure (“early” genes), and 86 more within 40 minutes (“late” genes; Figure 5D). In contrast, 62 genes requiring VNG0258H for repression in response to H_2_O_2_ form a single, coherent cluster, with no waves detected (Figure 5C). The third cluster includes 27 genes which show increased expression in *ΔVNG0258H* in the absence of H_2_O_2_ (Figure 5E). Finally, the fourth cluster includes 13 genes which exhibit altered dynamics in Δ*VNG0258H* (Figure 5F and G). These genes exhibited an impulse-like wave of expression in the parent strain. Although the expression patterns of these genes were equivalent in *ΔVNG0258H* and the parent for the first 40 minutes following H_2_O_2_ exposure, expression levels remained elevated compared to the parent level for the duration of the time course (Figure 5F). The converse pattern was also detected (Figure 5G). Across all four clusters combined, approximately equal proportions of the 294 VNG0258H-dependent genes are under-expressed (48%; Figure 5B, D, E, G) as are over-expressed (52%; Figure 5A, C, F) in *ΔVNG0258H*. Together these data suggest that VNG0258H (a) is bifunctional, required for the activation of some genes and the repression of others in response to H_2_O_2_ (Figure 5); and (b) may be involved in fine-tuning of gene expression dynamics for a subset of genes. 

#### Clusters of gene expression patterns in response to PQ

**Figure 6:**
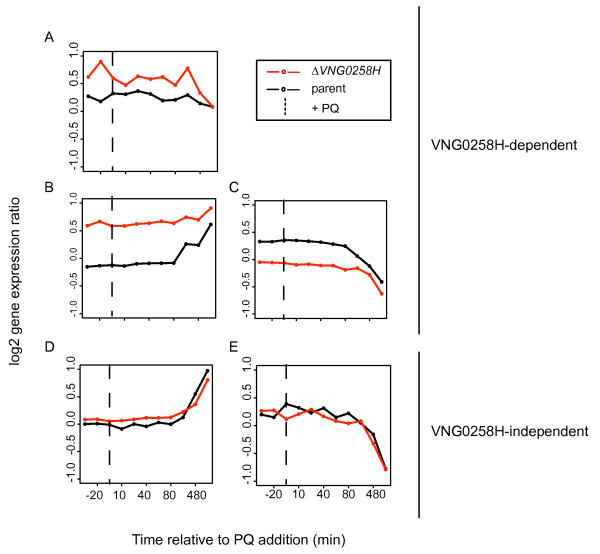
**Gene expression in response to PQ exposure in the Δ*ura3*parent*vs.*Δ*VNG0258H*mutant strains.** Line graphs represent mean expression profiles for each cluster of genes. Colors are as in Figure 5. The dotted line on each graph represents the time of PQ addition to each culture. (**A**) Genes requiring VNG0258H for repression regardless of PQ addition. (**B**) Genes requiring VNG0258H for repression in the absence of PQ and up-reguation in the presence of PQ. (**C**) Genes requiring VNG0258H for activation in the absence of PQ. (**D**) Genes induced in response to PQ but independent of VNG0258H. (**E**) Genes repressed in response to PQ but independent of VNG0258H. Gene expression profiles for individual genes in each cluster are shown in Additional file 9: Figure S4. Annotation details for genes in each cluster are listed in Additional file 5: Table S3.

The mRNA levels for 188 genes changed in abundance in response to PQ addition to mid-logarithmic phase cultures but exhibited similar dynamic patterns in the parent strain and Δ*VNG0258H*. This indicates that these genes do not rely on VNG0258H for their response to PQ (“VNG0258H-independent”; Additional file 5: Table S3, Figure 6D, E). In contrast, 61 genes were VNG0258H-dependent (Figure 6A, B, C). Of these 61, 7 genes were upregulated in Δ*VNG0258H* but unaffected by PQ in the Δ*ura3* parent strain (Figure 6A). 30 genes were up-regulated dynamically in the Δ*ura3* parent in response to PQ but were constitutively up-regulated in Δ*VNG0258H*, suggesting that VNG0258H is required to repress these genes during standard growth conditions and that this repression is relieved in response to PQ (Figure 6B). The remaining 24 genes were down-regulated in response to PQ in the parent but remained low through the duration of the experiment in the Δ*VNG0258H* strain (Figure 6B and C).

Upon comparison of the VNG0258H-dependent genes from the H_2_O_2_ experiment to those from the PQ experiment, we observed that 32 genes (50 including predicted operon members) were members of both lists, suggesting that these genes are dependent upon VNG0258H regardless of growth condition or stress treatment (Table 1). These genes are considered to be the core VNG0258H regulon.

**Table 1:** RosR regulon with arCOG data (archaeal Clusters of Orthologous Groups)

**ORF**	**Gene alias**	**arCOG ID**	**Category**	**Protein function**
VNG0144H	*VNG0144H*	arCOG02761	S	Uncharacterized conserved protein
VNG0255C	*VNG0255C*	arCOG02942	L	Ribonuclease HI
VNG0256H	*VNG0256H*	arCOG04769	S	Uncharacterized conserved protein
VNG0439C	*VNG0439C*	arCOG00570	C	Dehydrogenase (flavoprotein)
VNG0485H	*VNG0485H*	arCOG09222	S	Uncharacterized conserved protein
VNG0486G	*hat1*	arCOG00842	J	Acetyltransferase, RimL family
VNG0487H	*VNG0487H*	arCOG00842	J	Acetyltransferase, RimL family
VNG0488H	*VNG0488H*	arCOG04770	I	Acyl-CoA synthetase (AMP-forming)/AMP-acid ligase II
VNG0506H	*VNG0506H*	arCOG09224	S	Uncharacterized conserved protein
VNG0556G	*sgb*	arCOG02209	R	Polysaccharide biosynthesis protein, Mvin family
VNG0777G	*taqD*	arCOG01222	M	Cytidylyltransferase fused to conserved domain of DUF357 family
VNG0778C	*VNG0778C*	arCOG01139	R	Predicted metal-dependent protease of the PAD1/JAB1 superfamily
VNG1041H	*VNG1041H*		NA	NA
VNG1201G	*fucA*	arCOG04226	G	Fuculose-1-phosphate aldolase
VNG1202C	*VNG1202C*	arCOG02291	R	HAD superfamily hydrolase
VNG1204G	*gdhA2*	arCOG01352	E	Glutamate dehydrogenase/leucine dehydrogenase
VNG1246H	*VNG1246H*	arCOG04608	S	Uncharacterized conserved protein
VNG1330H	*VNG1330H*	arCOG07569	S	Uncharacterized conserved protein
VNG1332G	*sod2*	arCOG04147	P	Superoxide dismutase
VNG1343C	*VNG1343C*	arCOG04303	R	Uncharacterized Rossmann fold enzyme
VNG1404G	*trh1*	arCOG02815	K, O	Putative transcripion factor, Lrp family (K). Conserved domain frequently associated with peptide methionine sulfoxide reductase (O).
VNG1425H	*VNG1425H*	arCOG04789	S	Uncharacterized conserved protein
VNG1444G	*hisD*	arCOG04352	E	Histidinol dehydrogenase
VNG1474G	*est*	arCOG01648	R	Alpha/beta superfamily hydrolase
VNG1533H	*VNG1533H*	arCOG06229	S	Uncharacterized conserved protein
VNG1589C	*VNG1589C*	arCOG09277	S	Uncharacterized conserved protein
VNG1749G	*gbp1*	arCOG00357	J	Predicted GTPase, probable translation factor
VNG1948H	*VNG1948H*	arCOG04525	S	Uncharacterized conserved protein
VNG1963H	*VNG1963H*		NA	NA
VNG2184G	*tfbA*	arCOG01981	K	Transcription initiation factor TFIIIB, Brf1 subunit/Transcription initiation factor TFIIB
VNG2286G	*mamA*	arCOG01710	I	Methylmalonyl-CoA mutase, C-terminal domain/subunit (cobalamin-binding)
VNG2288G	*mamB*	arCOG06231	E	Glutamate mutase epsilon subunit
VNG2289G	*mal*	arCOG06232	E	Methylaspartate ammonia-lyase
VNG2290G	*maoC1*	arCOG00775	I	Acyl dehydratase
VNG2291G	*cat*	arCOG06124	C	Acetyl-CoA hydrolase
VNG2376H	*VNG2376H*	arCOG04728	S	Uncharacterized conserved protein
VNG2444C	*VNG2444C*	arCOG01141	R	Phosphoesterase
VNG2556H	*VNG2556H*	arCOG09321	S	Uncharacterized conserved protein
VNG2570G	*dcd*	arCOG04048	F	Deoxycytidine deaminase
VNG2591C	*VNG2591C*	arCOG02264	S	Predicted membrane protein
VNG2593H	*VNG2593H*	arCOG03026	O	Thioredoxin-like protein
VNG2594C	*VNG2594C*	arCOG09323	S	Uncharacterized conserved protein
VNG2669G	*cyo*	arCOG04471	S	Predicted membrane protein
VNG5143C	*VNG5143C*	arCOG09333	R	Predicted permease
VNG5157H	*VNG5157H*		NA	NA
VNG5164C	*VNG5164C*	arCOG04311	R	Predicted hydrolase of HD superfamily
VNG6275H	*VNG6275H*		NA	NA
VNG6276H	*VNG6276H*	arCOG09354	S	Uncharacterized conserved protein
VNG6312G	*argS*	arCOG00487	J	Arginyl-tRNA synthetase
VNG6313G	*nhaC3*	arCOG02010	C	Na+/H + antiporter

**Figure 7:**
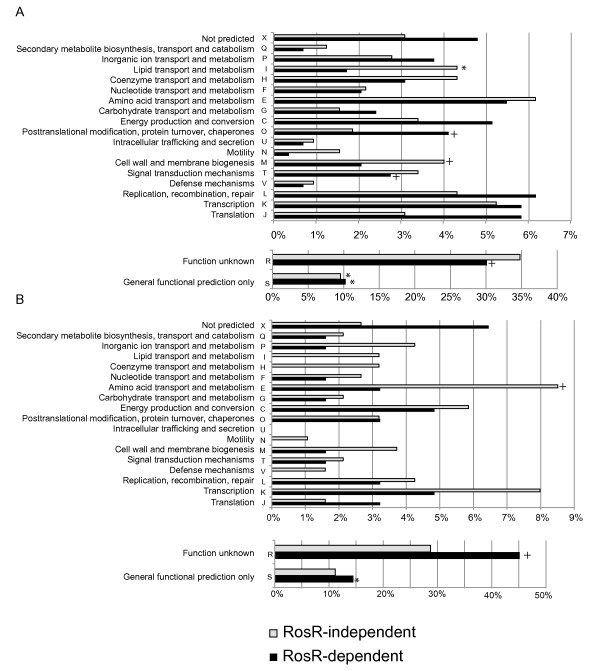
**Genes dependent on RosR and responsive to ROS (H**_**2**_**O**_**2**_**and PQ) are enriched for functions in protein and DNA repair.** (**A**) Predicted functions of genes differentially expressed in response to H_2_O_2_ according to archaeal Clusters of Orthologous Groups (arCOG) categories [48]. Category annotations are listed in on the Y-axis. Black bars represent the number of genes in each category dependent upon RosR for their differential expression (Figure 5A-G), whereas grey bars enumerate genes in each category that are differentially expressed in response to H_2_O_2_ but not affected by the Δ*rosR* mutation (*i.e.* “RosR-independent”, Figure 5H and J). (**B**) Predicted functions of genes differentially expressed in response to paraquat (PQ) according to arCOG. Colors and category annotations are as in (A). Asterisks denote significant overrepresentation of a functional category with *p* < 0.05. Plus signs (+) indicate enrichment *p* < 0.2. Details of gene annotations, membership in each arCOG category, and *p*-values for enrichment for all categories are listed in Additional file 4: Table S2 for H_2_O_2_ data and Additional file 5: Table S3 for PQ data.

ORF - open reading frame number from *H. salinarum* NRC-1 genome [44]; Gene alias – where present, common four-letter name of protein; arCOG ID, identification code for each arCOG sub-group annotation [48]; Category – arCOG category letter designation for each protein (as in Figure 7, Additional file 4: Table S2, Additional file 5: Table S3); protein function – function of predicted protein product as annotated by arCOGs; NA, not in arCOGs.

#### Functional enrichment in gene expression clusters

According to archaeal Clusters of Orthologous Groups (arCOG) categories, the 294 VNG0258H-dependent genes (Figure 5) were found to be 2-fold enriched for functions in protein turnover/chaperones (category O) compared to the 332 VNG0258H-independent genes (*p*-value < 0.2 *vs.* 0.96 for VNG0258H-independent genes, Additional file 4: Table S2). We also observed 2-fold enrichment in translation (category J) and 1.5-fold in DNA recombination and repair (category L) (Figure 7A). For example, two gene products in the cluster dependent upon VNG0258H for impulse-like dynamics (Figure 5F and G) are predicted to function in DNA mismatch repair (*i.e. mutS1, mutT*) and one as a TF (*i.e. VNG0704C*; Figure 7A, Additional file 4: Table S2). The majority of targets were of unknown function (Figure 7A; *p* < 0.025).

Genes dependent upon VNG0258H for differential expression in response to paraquat are mostly of unknown function (Figure 7B; *p* < 2.5 x 10^-4^), though they are also enriched for genes predicted to be involved in translation (Figure 7B). Genes that are VNG0258H-dependent in both PQ and H_2_O_2_ stress conditions have varied functions (Table 1), including transcriptional regulation (*tfbA* and Lrp-like regulator *trh1*), superoxide detoxification (*sod2*), and amino acid metabolism (*e.g.* histidine and arginine biosynthesis).

Surprisingly, we also detected functional enrichment for lipid transport and metabolism (category I, *p* < 0.05) and cell wall biogenesis (category M, *p* = 0.0515) for genes that are responsive to H_2_O_2_ but independent of VNG0258H regulation. We did not detect these enrichments in response to PQ. Together these functional enrichments suggest that (a) VNG0258H plays an important role in the regulation of protein production and/or turnover and DNA repair systems as well as currently uncharacterized cellular processes; and (b) lipid metabolism and cell wall biogenesis functions may be important in the specific response to H_2_O_2_ in this organism. In sum, the H_2_O_2_ and PQ gene expression data (Figure 5, Figure 6, Additional file 8: Figure S3 and Additional file 9: Figure S4, Additional file 4: Table S2 and Additional file 5: Table S3) suggest that VNG0258H is a bifunctional regulator of genes whose products are required for ROS resistance. VNG0258H may be required for impulse-like dynamics and time-resolved waves of gene expression in response to H_2_O_2_ but not PQ. We will therefore henceforth refer to VNG0258H as RosR, or *r*eactive *o*xygen *s*pecies *r*egulator.

### RosR binds directly to the chromosomal locus encoding superoxide dismutase

**Figure 8:**
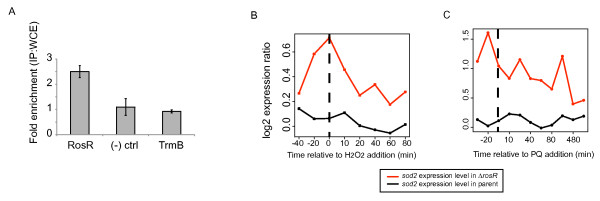
**ChIP-qPCR data suggest that RosR binds DNA directly.** Relative enrichment ratio is shown for a 100-bp region in the *sod2* promoter region in immunoprecipitates (IP) compared to randomly sheared chromosomal DNA (whole cell extract, WCE). Enrichments are compared for the putative VNG0258H transcription factor, empty plasmid (“mock”) and TrmB (a transcription factor known not to bind to the *sod2* locus [20]). Error bars represent +/− SEM from the mean of 4 biological replicate experiments. (**B**) *sod2* (superoxide dismutase) is overexpressed in Δ*rosR vs.* the parent strain in H_2_O_2_ gene expression experiments. Red trace represents microarray gene expression data (also shown in Figure 5A) for *sod2* in Δ*rosR*, whereas the black trace is for the Δ*ura3* parent strain. (**C**) *sod2* (superoxide dismutase) is overexpressed in Δ*rosR* vs the parent strain in PQ gene expression experiments. Colors are as in (B).

To determine if RosR’s effects on gene expression are mediated *via* direct interaction with DNA, we performed *in vivo* binding analysis using chromatin immunoprecipitation (ChIP [29]) coupled to quantitative PCR (qPCR [27]). We detected direct RosR-DNA binding to the *sod2* locus, whose product functions as a manganese-binding superoxide dismutase in *H. salinarum*[11]. The *sod2* transcript is also significantly activated in Δ*rosR* regardless of which oxidant is added (Figure 8B8C, Table 1). Under standard conditions (mid-log phase, rich medium, 37°C), the *sod2* locus is 2.5-fold enriched for binding to RosR over the controls, which included mock input and TrmB transcription factor (previously shown not to bind the *sod2* locus, [20]; Figure 8A). These results demonstrate that RosR binds to DNA under standard growth conditions. Combined with the gene expression microarray experiments (Figures 5 and 6), these data suggest that RosR-DNA binding is associated with repression of *sod2* transcriptional activity. 

### Refining the gene regulatory network

To assess the accuracy of the GRN predictions [11], we compared the gene expression results described here (Figures 5 and 6) to the predictions of the GRN (Figure 1). Predictions from the model suggested that RosR regulates cobalamin biosynthesis (*cbiJ,* gene set 91, Figure 1) and oxidoreductase genes (*yajO2,* gene set 6, Figure 1), which our H_2_O_2_ gene expression results have confirmed (Figure 5C, Additional file 4: Table S2). However, the prediction that RosR regulates genes involved in iron homeostasis (e.g. siderophore uptake genes *iucABC*; gene set 12) was not confirmed here (i.e. the expression of these genes were not significantly affected by the Δ*rosR* deletion).

We also explored the GRN for cis-regulatory sequence predictions. Of the three sets of genes that were predicted to be RosR-dependent (set 6, 12, and 91, Figure 1), only set 12 contained a cis-regulatory sequence prediction [11]. Therefore, we conducted *de novo* motif discovery using the MEME algorithm (see Methods). We conducted two different computational searches, including (a) phylogenetic footprinting [24] with the *sod2* promoter sequences from all haloarchaeal genomes with a predicted RosR homolog (Figure 2), and (b) searches using promoters sequences of all 50 genes shared between the PQ and H_2_O_2_ datasets (Table 1). Using MEME, we detected a set of related putative motifs, each of which has a high likelihood of containing a central palindromic TCG-N-CGA motif (*p* < 7 x 10^-56^, Additional file 10: Figure S5) flanked by consensus sequences of varying strength. Taken together, the gene expression and putative cis-regulatory sequence results described here confirm and refine the statistically inferred GRN prediction [11].

## Discussion

Here we have used a systems biology approach to identify and characterize a novel transcription factor, RosR. This protein is required for survival in the face of extremely high levels of ROS exposure (Figures 3 and 4), as it activates and represses genes encoding macromolecule repair and cellular maintenance functions (Figures 5, 6, 7). It directly binds the promoter of *sod2* (Figure 8). Although future studies are necessary to differentiate whether the remaining genes are direct or indirect targets of RosR, these results support the conclusion that RosR may be a bifunctional transcription factor that regulates the extreme ROS response of *H. salinarum.*

*H. salinarum* grows at remarkably high PQ and H_2_O_2_ stress conditions [9,11] compared to ROS-sensitive species such as *E. coli*[3], which the results reported here corroborate (Figures 3,4). For example, we found that more than 0.3 mM PQ is required to decrease *H. salinarum Δura3* growth rate to half its original rate, whereas it takes only about 0.1 mM PQ to achieve a comparable decrease in growth rate in *E. coli* B [53]. Similarly, 1 mM H_2_O_2_ is lethal to *E. coli*[54], whereas *H. salinarum* survives up to 25 mM H_2_O_2_.

Similar to *E. coli* and other mesophiles, H_2_O_2_ resistance in *H. salinarum* is proportional to cell density, whereas PQ resistance is independent of cell number (Figures 3 and 4, [55]). Previous studies in *E. coli* suggest that H_2_O_2_ scavenging capacity is higher in dense cultures due to increased concentration of scavenging enzymes [56]. In contrast, PQ is a redox cycling drug that continually produces endogenous ROS in the cell membrane and so cannot be cleared from the culture during growth [57]. This difference in chemistry of the oxidants and the different responses of *H. salinarum* observed here suggest that PQ may be a better proxy for ROS damage resulting from continuous UV exposure in the salt lake environment.

*H. salinarum* uses a battery of enzymatic and non-enzymatic strategies to withstand macromolecular damage in its highly oxidative, saturated salt habitat, including genetic redundancy of DNA repair and antioxidant enzyme-coding genes [4,6,7]. Interestingly, among the functionally redundant DNA repair genes MutT and MutS, we found that RosR regulates only one of each of the paralogs (*e.g. mutS1* and not *mutS2*/*3*). This suggests that the function of enzymes encoded by these genes could be only partially redundant. Alternatively, dynamic regulation of each may contribute to differential timing of expression and function. In contrast, both superoxide dismutase genes (*sod1* and *sod2*) are differentially expressed in Δ*rosR* in response to H_2_O_2_ (Additional file 4: Table S2). Combined with the growth data results that the Δ*rosR* mutant growth defect is most dramatic during exposure to high concentrations of PQ and H_2_O_2_ (Figures 3 and 4), our findings suggest that RosR regulation represents another important component of the mechanism for ROS protection and repair in this environment and, by homology, perhaps also in other haloarchaea.

Our results suggest that RosR may play additional roles in cellular physiology. A large proportion of the genes dependent upon RosR for appropriate differential expression are of unknown function (30% of genes in response to H_2_O_2_ and 45% in response to PQ, Figure 7). In addition, RosR activates and represses genes that are independent of oxidant treatment (Figures 5A, B and 6A). In previous studies, many of these genes were also induced in response to other conditions that damage macromolecules (e.g. UV and gamma radiation, [2]). In addition, although no growth defect was observed under standard aerobic growth conditions (Figures 3 and 4), it remains formally possible that RosR is involved in regulating gene expression in response to oxygen shifts in cooperation with other TFs, especially given the strong correlation of the *rosR* gene expression profile with oxygen shifts (Figure 1D). Future work will investigate the role of RosR in the response to such conditions.

**Figure 9:**
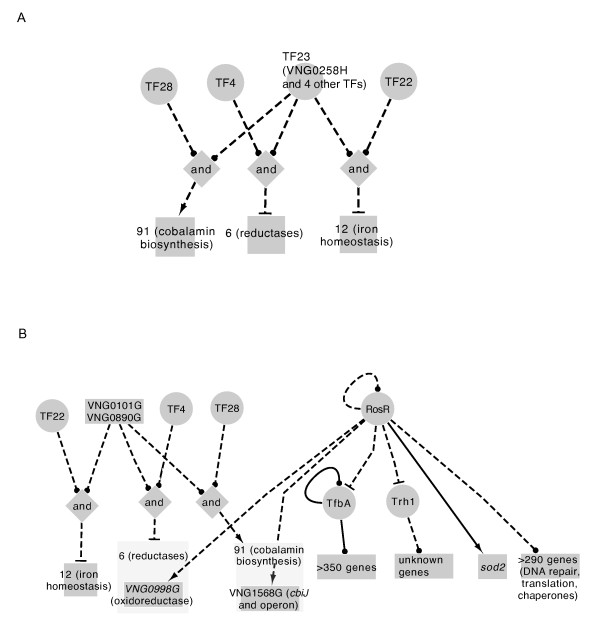
**Comparison of GRN topology from previous studies with the RosR regulon characterized here.** (**A**) Subnetwork diagram depicting predictions regarding putative VNG0258H function from the ROS environmental gene regulatory inference network (EGRIN) (adapted from [11]). Circles represent transcription factor (TF) groups. TF group 23 includes VNG0258H and four other putative TFs (VNG0101G, VNG0347G, VNG1496G, and VNG0890G). Diamonds represent combinatorial logic gates (AND). Blunt arrows represent computationally inferred repression influences, whereas pointed arrows are activation influences. Squares represent inferred clusters of co-regulated genes and numbers within the squares refer to EGRIN cluster IDs. (**B**) Refinements to GRN model based on the work presented in the current study. Node shapes and edge attributes are as in (A). Solid lines indicate direct regulation (*i.e.* DNA-protein interaction has been detected), whereas dotted lines represent interactions that could be direct or indirect (*i.e.* direct interaction still needs to be tested). Dark grey boxes within light grey boxes indicate that, out of all genes in the cluster, only the gene indicated in dark grey is RosR-dependent based on the microarray data from the current study.

Other TFs are likely to be involved in the ROS response in *H. salinarum*. Our gene expression data suggest that genes previously implicated in ROS protection and damage repair in this organism do not require RosR in response to H_2_O_2_ and PQ shock (e.g. non-homologous end joining and base excision repair pathways, thioredoxin, and catalase [11]; Figure 5H and I, Figure 6D and E, Figure 7). Candidate TFs for such regulation include VNG0101G, VNG0347G, VNG1496G, and VNG0890G, which are nearest neighbors to RosR in the existing network (Figure 9) [11]; or the Trh1 and TfbA transcription factors, whose corresponding genes require RosR for appropriate expression (Table 1). 

Our results refine the previously published GRN model [11]. According to the model, RosR is combined within the same regulatory node with four other TFs (Figure 9A). Together, these genes are predicted to influence the expression of genes involved in oxidative stress repair and metal homeostasis, cobalamin biosynthesis, and redox reactions. Here we have differentiated which genes included in this prediction are RosR-dependent and which may be dependent on the four other TFs (Figure 9B). We have also added cis-regulatory sequence predictions that were missing from the initial model. Although the predicted cis-regulatory sequence detected in *sod2* promoters is relatively degenerate (i.e. only 6 nt long), the conservation of a putative cis-regulatory sequence in the promoter of *sod2* with those from other haloarchaea is consistent with the idea of an evolutionarily conserved RosR function (Additional file 10: Figure S5). Second, we observed that RosR is required for impulse-like, time-resolved waves of gene expression in response to H_2_O_2_ (Figure 5). Previous theoretical studies suggest that such dynamics could result either from autoregulatory feedback or feed-forward loops comprised of two TFs [58]. Thus, RosR could regulate itself or work in concert with other transcription factor(s) (see candidates above) to bring about impulse-like dynamics.

RosR is highly conserved among haloarchaeal species but poorly conserved among other archaea and bacteria (Figure 2). Indeed, four other paralogs of RosR (E-value < 5 x 10^-19^) are present in the genome of *H. salinarum* alone [32]. In other archaea, only one other ROS-specific transcription factor, MsvR in methanogens, has been identified and characterized [24]. Like RosR, MsvR also appears to be restricted to a small subset of species [24] and functions to repress oxidative stress genes, suggesting interesting evolutionary questions. Sulfonylation [52] or oxidation of cysteine residues [57,59] is the primary mechanism for conformational changes of redox-responsive transcription factors in bacteria and has been hypothesized for MsvR. These conformational changes influence interactions with DNA. The RosR protein lacks cysteine residues and other typical sequences in the effector domains (Figure 2), so the biochemical mechanism by which RosR binds DNA and senses oxidants remains unclear.

## Conclusions

We conclude that RosR is a haloarchaeal-specific, wHTH transcription factor important for gene regulation in response to highly oxidative conditions. We further suggest that RosR is an important node in a large, interconnected gene regulatory network (GRN) regulating the response to oxidative stress. This study lays groundwork for understanding how the haloarchaea may have evolved to thrive in their extremely oxidative, hypersaline niche.

## Competing interests

The authors declare that they have no competing interests.

## Authors’ contributions

KS performed all growth experiments and analyzed all high throughput growth data. KS performed all microarray experiments. NG wrote and implemented the microarray normalization and preprocessing pipeline, calculated enrichments in arCOG functional categories, and assisted with microarray data analysis. JLB performed ChIP-qPCR experiments and analyzed the data. AS analyzed all existing systems biology datasets, new RosR microarray data, and performed data integration and interpretation. AS and KS wrote the paper, whereas NG and JLB edited the paper. All authors have read and approved the final manuscript.

## Supplementary Material

Additional file 1 **Table S4.** Lists primers and strains used in this study. Click here for file

Additional file 2 Supplementary Methods & Results. Click here for file

Additional file 3 **Table S1.** Includes raw and analyzed cell density data (as OD600 values) from each growth curve experiment in the Bioscreen C instrument (main text Figures 3 and 4, Additional file 6: Figure S1 and Additional file 7: Figure S2). Please see legends for information regarding each section of the Table. Click here for file

Additional file 4 **Table S2.** All gene expression microarray data, annotation details, and arCOG memberships for each gene cluster from main text Figure 5 (H_2_O_2_ exposure) are listed. Please see the tab labeled “legend” for information regarding each section of the Table. Click here for file

Additional file 5 **Table S3.** Gene expression microarray data and arCOG functional annotations for paraquat (PQ) gene expression data. Please see the tab labeled “legend” for information regarding each section of the Table. Click here for file

Additional file 6 **Figure S1.** Growth in batch culture is similar to that in the Bioscreen C. (A) Top: comparison of growth yield under standard conditions (*i.e.* no stress) in batch *vs.* Bioscreen C. Δ*ura3* and Δ*VNG0258H* maximum cell density (OD600) are shown for the mean of 5 biological replicate samples with 2 technical replicates each. Error bars represent standard deviation from the mean. Bottom: comparison of growth rates under standard conditions in batch culture *vs.* Bioscreen. Columns and error bars are as in (A). (C) Representative growth curves for Δ*ura3* parent strain and *ΔVNG0258H* mutant strains in response to H_2_O_2_ added in mid-logarithmic phase in batch culture. Addition of H_2_O_2_ indicated by arrow. Cell density (OD600) was measured in a standard spectrophotometer at the times indicated. Strains and conditions are indicated in the *legend*. (D) Representative growth curves in batch culture under paraquat (PQ) conditions. Click here for file

Additional file 7 **Figure S2.** A wild type copy of the *VNG0258H* gene supplied on a plasmid (pMTFcmyc vector, [1]) complements the Δ*VNG0258H* growth defects. (A) Box-whisker plots depicting growth rates of *H. salinarum* strains in the bioscreen C (*Δura3* parent, *ΔVNG0258H* mutant, and *ΔVNG0258H* mutant complemented *in trans*) during the 12 hours following H_2_O_2_ shock (mid-logarithmic phase addition of H_2_O_2_). Horizontal lines within each box represent the median growth rate across 24 replicate trials (8 biological replicates, 3 technical replicates) for each strain in each condition. Boxes represent the interquartile range (IQR), and whiskers are minimum and maximum values within 1.5 times the IQR. Concentrations of H_2_O_2_ added are indicated on the X-axis, whereas the Y-axis quantifies growth rate. (B) Box-whisker plot depicting lag phase addition of H_2_O_2_ to Bioscreen cultures. Boxes, median lines, and whiskers are as in (A). Y-axis expresses the growth rate of the Δ*VNG0258H* or trans-complemented strains as a function of Δ*ura3* growth rate. (C) Box-whisker plot depicting survival ratios 24 hours after mid-logarithmic phase addition of 25 mM H_2_O_2_ to batch cultures. (D) Box-whisker plot depicting growth rates following mid-logarithmic phase addition of PQ to batch cultures. Growth rates are expressed as a function of Δ*ura3* parent strain growth. Click here for file

Additional file 8 **Figure S3.** Detailed heat map for each gene cluster from main text Figure 5 (H_2_O_2_ exposure). Data for those genes dependent on VNG0258H for appropriate expression are shown (i.e. main text Figures 5A-G). Gene names are listed on the right of each heat map. Detailed annotations and COG category memberships (main text Figure 7A) for each these genes are listed in Additional file 4: Table S2. In each heat map, red represents induction, whereas blue represents repression. VNG0258H-independent genes (Cluster 4, Figure 5H and J) are not included in the Figure for brevity and clarity, but expression data and annotations for these genes are included in Additional file 4: Table S2. (A) Cluster 1 includes genes that were differentially expressed in the Δ*VNG0258H* mutant vs Δ*ura3* parent strain regardless of growth condition (main text Figures 5A-B). Cluster 1a (main text Figure 5A) depicts those 33 genes that are over-expressed in the Δ*VNG0258H* mutant (i.e. RosR is required to repress these genes). Cluster 1b (main text Figure 5B) depicts those 30 genes that are under-expressed in the Δ*VNG0258H* mutant (i.e. VNG0258H is required to activate these genes). (B) Cluster 2 includes genes that were differentially expressed in the Δ*VNG0258H* mutant vs Δ*ura3* parent strain in the presence of H_2_O_2_ (main text Figures 5C-D). Cluster 2a (main text Figure 5C) contains those 43 genes that are over-expressed in the Δ*VNG0258H* mutant in response to H_2_O_2_ (i.e. VNG0258H is required to repress these genes in response to H_2_O_2_). Cluster 2b (main text Figure 5D) contains those genes that are under-expressed in the Δ*VNG0258H* mutant in response to H_2_O_2_ (i.e. VNG0258H is required to induce them). (C) Cluster 3 includes genes that were differentially expressed in the Δ*VNG0258H* mutant vs Δ*ura3* parent strain in the absence of H_2_O_2_ (main text Figure 5E). (D) Growth of Δ*ura3* parent and Δ*VNG0258H* cultures for gene expression microarray analysis. Black curves represent growth data for the two biological replicate cultures of Δ*ura3*, whereas red curves are data for the two biological replicate cultures of Δ*VNG0258H*. Dotted arrows on the curves indicate the start and end of sampling over the time courses shown in the heat maps, whereas the solid arrow shows the time of H_2_O_2_ addition to the cultures. Click here for file

Additional file 9 **Figure S4.** Detailed heat map for each gene cluster from main text Figure 6. Data for those genes dependent on RosR for appropriate expression in response to PQ are shown (main text Figures 6A-C). Colors and labels are as in Additional file 8: Figure S3. (A) Heatmap for Cluster 1, genes differentially expressed in *ΔrosR* vs the *Δura3* parent strain regardless of growth condition (main text Figure 6A). (B) Heatmap for Cluster 2, genes dependent upon RosR for differential expression in response to paraquat (PQ). Genes upregulated in the mutant are shown on the left (main text Figure 6B) and those downregulated are shown on the right (main text Figure 6C). (C) Genes differentially expressed in response to PQ that are independent of RosR. Upregulated genes are shown (main text Figure 6D). Downregulated genes (171 genes) are not shown for brevity, but are listed in Additional file 5: Table S3. (D) Growth data for cultures from which RNA was harvested for microarray studies. Red arrow indicates the time of PQ addition. Click here for file

Additional file 10 **Figure S5.** Putative cis-regulatory sequences resulting from MEME analysis on (A) the 50 genes differentially expressed in common in the PQ and H2O2 gene expression datasets (main text Table 1), and (B) phylogenetic footprinting using ***sod2*** promoter sequences from all halophilic archaea possessing a RosR homolog. Each sequence logo represents a different ***cis*** sequence prediction. The height of the letters in each nucleotide position represents the strength of the consensus between the input sequences. The putative TCG-N-CGA motif is boxed in each case. In (A), the top-scoring two motifs from MEME searches are shown. Top motif *p*-value is 7.0x10^-56^, and bottom motif *p*-value is 2.6x10^-42^. 43 of the 50 promoter query sequences contained each motif. In (B), only the top-scoring motif is shown. Click here for file
