# Supplementary material for: The RosR transcription factor is required for gene expression dynamics in response to extreme oxidative stress in a hypersaline-adapted archaeon
Source: BMC Genomics. 2012 Jul 30;13:351. doi: 10.1186/1471-2164-13-351 (PMC3443676; doi:10.1186/1471-2164-13-351)
Supplement: Additional file 2 — Supplementary Methods & Results. [file 1471-2164-13-351-S2.doc]

**GUIDE TO SUPPLEMENTARY MATERIAL**

**SUPPLEMENTARY MATERIAL 1. Details regarding growth assays.**

**Supplementary Methods.** *∆ura3* and *∆VNG0258H* mutant cultures were grown to mid-logarithmic phase in batch culture to compare to growth in high throughput in the Bioscreen C. Each strain was challenged with 25 mM H_2_O_2_ or 0.3 mM paraquat (PQ). Growth rates were calculated for batch culture in the same way as for Bioscreen (see Methods, main text). Overall growth yield was calculated by averaging the OD600 from stationary phase time points. By these metrics, we conclude that growth in the Bioscreen C is not significantly different from growth in batch culture (Supplementary Figure 1A and B). The growth defect of the ∆*VNG0258H* strain in H_2_O_2_ and PQ observed in Bioscreen experiments was recapitulated in batch culture (Supplementary Figure 1C and D).

In order to add oxidant (H_2_O_2_ or PQ) to Bioscreen C cultures in log phase, culture plates were removed from the instrument. OD_600_ readings recorded just after plates were replaced into the Bioscreen C tended to be artificially very low or very high. This was due to the condensation of liquid on the top and sides of plate wells when plates were moved from 37°C to room temperature, and due to the instrument incubator and bulb having cooled slightly during the period when plates were removed.

To compensate for these artifacts in the growth curve data, we manually smoothed a small portion of the representative curves shown in Figures 3D and 4C, where H_2_O_2_ or PQ was added in log phase. OD_600_ values that were gathered up to 1 hour after removal of a given plate from the Bioscreen C were replaced with values from a linear interpolation. These artifacts did not affect growth rate calculations, as growth rates were calculated beginning 1 hour after the addition of oxidant.

**Supplementary Figure 1.** Growth in batch culture is similar to that in the Bioscreen C. (A) Top: comparison of growth yield under standard conditions (*i.e.* no stress) in batch *vs.* Bioscreen C. ∆*ura3* and ∆*VNG0258H* maximum cell density (OD600) are shown for the mean of 5 biological replicate samples with 2 technical replicates each. Error bars represent standard deviation from the mean. Bottom: comparison of growth rates under standard conditions in batch culture *vs.* Bioscreen. Columns and error bars are as in (A). (C) Representative growth curves for ∆*ura3* parent strain and *∆VNG0258H* mutant strains in response to H_2_O_2_ added in mid-logarithmic phase in batch culture. Addition of H_2_O_2_ indicated by arrow. Cell density (OD600) was measured in a standard spectrophotometer at the times indicated. Strains and conditions are indicated in the *legend*. (D) Representative growth curves in batch culture under paraquat (PQ) conditions.

**Supplementary Figure 2.** A wild type copy of the *VNG0258H* gene supplied on a plasmid (pMTFcmyc vector, [1]) complements the ∆*VNG0258H* growth defects.

1. Box-whisker plots depicting growth rates of *H. salinarum* strains in the bioscreen C (*∆ura3* parent, *∆VNG0258H* mutant, and *∆VNG0258H* mutant complemented *in trans*) during the 12 hours following H_2_O_2_ shock (mid-logarithmic phase addition of H_2_O_2_). Horizontal lines within each box represent the median growth rate across 24 replicate trials (8 biological replicates, 3 technical replicates) for each strain in each condition. Boxes represent the interquartile range (IQR), and whiskers are minimum and maximum values within 1.5 times the IQR. Concentrations of H_2_O_2_ added are indicated on the X-axis, whereas the Y-axis quantifies growth rate.
2. Box-whisker plot depicting lag phase addition of H_2_O_2_ to Bioscreen cultures. Boxes, median lines, and whiskers are as in (A). Y-axis expresses the growth rate of the ∆*VNG0258H* or trans-complemented strains as a function of ∆*ura3* growth rate.
3. Box-whisker plot depicting survival ratios 24 hours after mid-logarithmic phase addition of 25 mM H_2_O_2_ to batch cultures.
4. Box-whisker plot depicting growth rates following mid-logarithmic phase addition of PQ to batch cultures. Growth rates are expressed as a function of ∆*ura3* parent strain growth.

**Supplementary Table 1** includes raw and analyzed cell density data (as OD_600_ values) from each growth curve experiment in the Bioscreen C instrument (main text Figures 3 and 4, and Supplementary Figures 1 and 2). Please see legends for information regarding each section of the Table.

**SUPPLEMENTARY MATERIAL 2. VNG0258H regulates gene expression in response to H_2_O_2_.**

**Supplementary Figure 3.** Detailed heat map for each gene cluster from main text Figure 5. Data for those genes dependent on VNG0258H for appropriate expression are shown (i.e. main text Figures 5A-G). Gene names are listed on the right of each heat map. Detailed annotations and COG category memberships (main text Figure 7A) for each these genes are listed in Supplementary Table 2. In each heat map, red represents induction, whereas blue represents repression. VNG0258H-independent genes (Cluster 4, Figure 5H and J) are not included in the Figure for brevity and clarity, but expression data and annotations for these genes are included in Supplementary Table 2.

1. Cluster 1 includes genes that were differentially expressed in the ∆*VNG0258H* mutant vs ∆*ura3* parent strain regardless of growth condition (main text Figures 5A-B). Cluster 1a (main text Figure 5A) depicts those 33 genes that are over-expressed in the ∆*VNG0258H* mutant (i.e. RosR is required to repress these genes). Cluster 1b (main text Figure 5B) depicts those 30 genes that are under-expressed in the ∆*VNG0258H* mutant (i.e. VNG0258H is required to activate these genes).
2. Cluster 2 includes genes that were differentially expressed in the ∆*VNG0258H* mutant vs ∆*ura3* parent strain in the presence of H_2_O_2_ (main text Figures 5C-D). Cluster 2a (main text Figure 5C) contains those 43 genes that are over-expressed in the ∆*VNG0258H* mutant in response to H_2_O_2_ (i.e. VNG0258H is required to repress these genes in response to H_2_O_2_). Cluster 2b (main text Figure 5D) contains those genes that are under-expressed in the ∆*VNG0258H* mutant in response to H_2_O_2_ (i.e. VNG0258H is required to induce them).
3. Cluster 3 includes genes that were differentially expressed in the ∆*VNG0258H* mutant vs ∆*ura3* parent strain in the absence of H_2_O_2_ (main text Figure 5E).
4. Growth of ∆*ura3* parent and ∆*VNG0258H* cultures for gene expression microarray analysis. Black curves represent growth data for the two biological replicate cultures of ∆*ura3*, whereas red curves are data for the two biological replicate cultures of ∆*VNG0258H*. Dotted arrows on the curves indicate the start and end of sampling over the time courses shown in the heat maps, whereas the solid arrow shows the time of H_2_O_2_ addition to the cultures.

**Supplementary Table 2.** All gene expression microarray data, annotation details, and arCOG memberships for each gene cluster from main text Figure 5 are listed. Please see the tab labeled “legend” for information regarding each section of the Table.

**SUPPLEMENTARY MATERIAL 3. RosR regulates gene expression in response to PQ.**

Unlike the immediate gene expression dynamics observed in response to H_2_O_2_, PQ-dependent changes in gene expression were not observed until two hours after the addition of PQ to mid-log phase cultures (Figure 6, Supplementary Figure 3), a response which continued for the duration of the 24 h time course. Because cultures began to transition into stationary phase at four hours after PQ addition, we reasoned that the observed gene expression changes could be a result of growth physiology rather than a specific PQ response. To explore this possibility, we compared the set of 188 PQ-responsive genes to those that are differentially expressed in response to stationary phase transition in the absence of PQ [2]. We detected that only 20 of the 188 PQ-responsive genes in our dataset were growth responsive. Of these 20, 17 were expressed in the opposite direction in the growth dataset vs PQ dataset. These results suggest that the differential expression observed here is specific to PQ rather than a growth effect.

**Supplementary Figure 4.** Detailed heat map for each gene cluster from main text Figure 6. Data for those genes dependent on RosR for appropriate expression in response to PQ are shown (main text Figures 6A-C). Colors and labels are as in Supplementary Figure 3. (A) Heatmap for Cluster 1, genes differentially expressed in *∆rosR* vs the *∆ura3* parent strain regardless of growth condition (main text Figure 6A). (B) Heatmap for Cluster 2, genes dependent upon RosR for differential expression in response to paraquat (PQ). Genes upregulated in the mutant are shown on the left (main text Figure 6B) and those downregulated are shown on the right (main text Figure 6C). (C) Genes differentially expressed in response to PQ that are independent of RosR. Upregulated genes are shown (main text Figure 6D). Downregulated genes (171 genes) are not shown for brevity, but are listed in Supplementary Table 3. (D) Growth data for cultures from which RNA was harvested for microarray studies. Red arrow indicates the time of PQ addition.

**Supplementary Table 4** lists primers and strains used in this study.

**Suplementary Figure 5.** Putative cis-regulatory sequences resulting from MEME analysis on (A) the 50 genes differentially expressed in common in the PQ and H2O2 gene expression datasets (main text Table 1), and (B) phylogenetic footprinting using *sod2* promoter sequences from all halophilic archaea possessing a RosR homolog. Each sequence logo represents a different *cis* sequence prediction. The height of the letters in each nucleotide position represents the strength of the consensus between the input sequences. The putative TCG-N-CGA motif is boxed in each case. In (A), the top-scoring two motifs from MEME searches are shown. Top motif *p*-value is 7.0x10^-56^, and bottom motif *p*-value is 2.6e-42. 43 of the 50 promoter query sequences contained each motif. In (B), only the top-scoring motif is shown.

**REFERENCES**

1. Facciotti MT, Reiss DJ, Pan M, Kaur A, Vuthoori M, Bonneau R, Shannon P, Srivastava A, Donohoe SM, Hood LE *et al*: **General transcription factor specified global gene regulation in archaea**. *Proc Natl Acad Sci U S A* 2007, **104**(11):4630-4635.

2. Facciotti MT, Pang WL, Lo FY, Whitehead K, Koide T, Masumura K, Pan M, Kaur A, Larsen DJ, Reiss DJ *et al*: **Large scale physiological readjustment during growth enables rapid, comprehensive and inexpensive systems analysis**. *BMC Syst Biol* 2010, **4**(64):64.
