# Supplementary figures and images for: The RosR transcription factor is required for gene expression dynamics in response to extreme oxidative stress in a hypersaline-adapted archaeon
Source: BMC Genomics. 2012 Jul 30;13:351. doi: 10.1186/1471-2164-13-351 (PMC3443676; doi:10.1186/1471-2164-13-351)

Supplementary Figure 1.

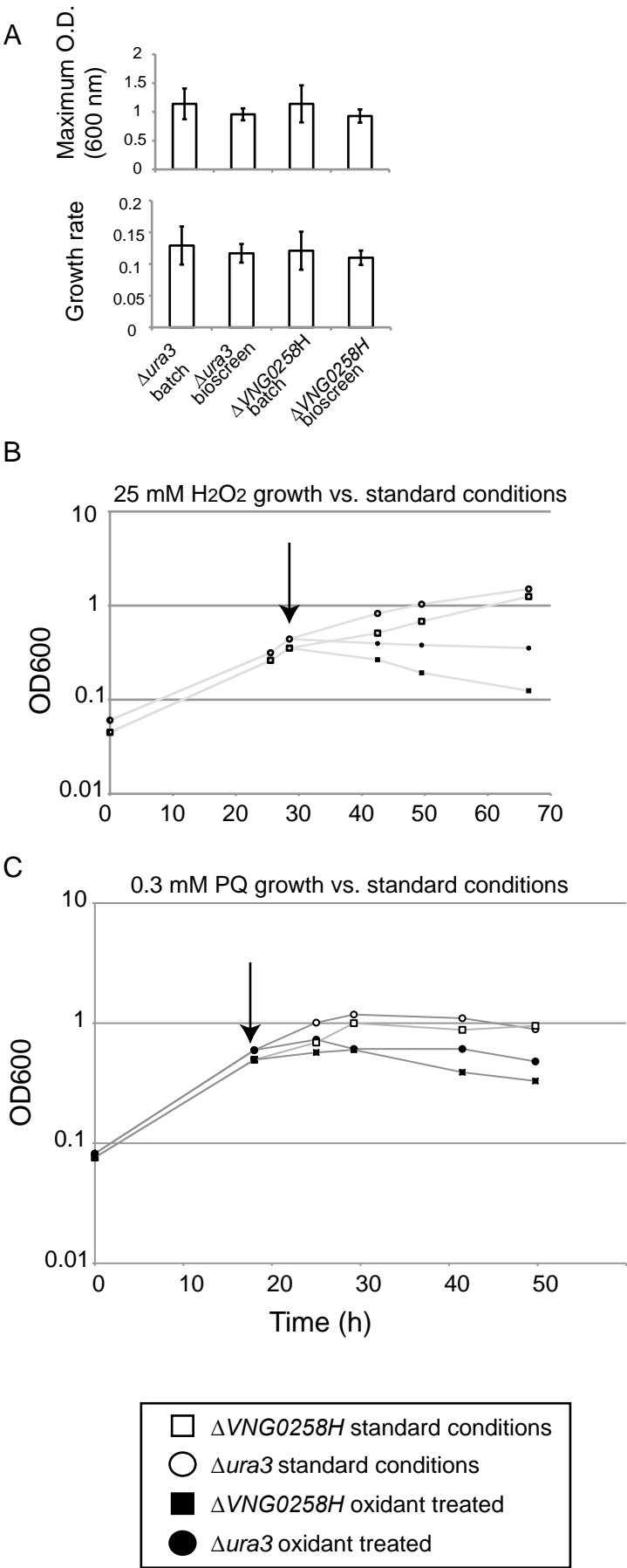

Supplement: Additional file 6 — Figure S1. Growth in batch culture is similar to that in the Bioscreen C. (A) Top: comparison of growth yield under standard conditions (i.e. no stress) in batch vs. Bioscreen C. Δura3 and ΔVNG0258H maximum cell density (OD600) are shown for the mean of 5 biological replicate samples with 2 technical replicates each. Error bars represent standard deviation from the mean. Bottom: comparison of growth rates under standard conditions in batch culture vs. Bioscreen. Columns and error bars are as in (A). (C) Representative growth curves for Δura3 parent strain and ΔVNG0258H mutant strains in response to H2O2 added in mid-logarithmic phase in batch culture. Addition of H2O2 indicated by arrow. Cell density (OD600) was measured in a standard spectrophotometer at the times indicated. Strains and conditions are indicated in the legend. (D) Representative growth curves in batch culture under paraquat (PQ) conditions. [file 1471-2164-13-351-S6.pdf]

Supplementary Figure 2.

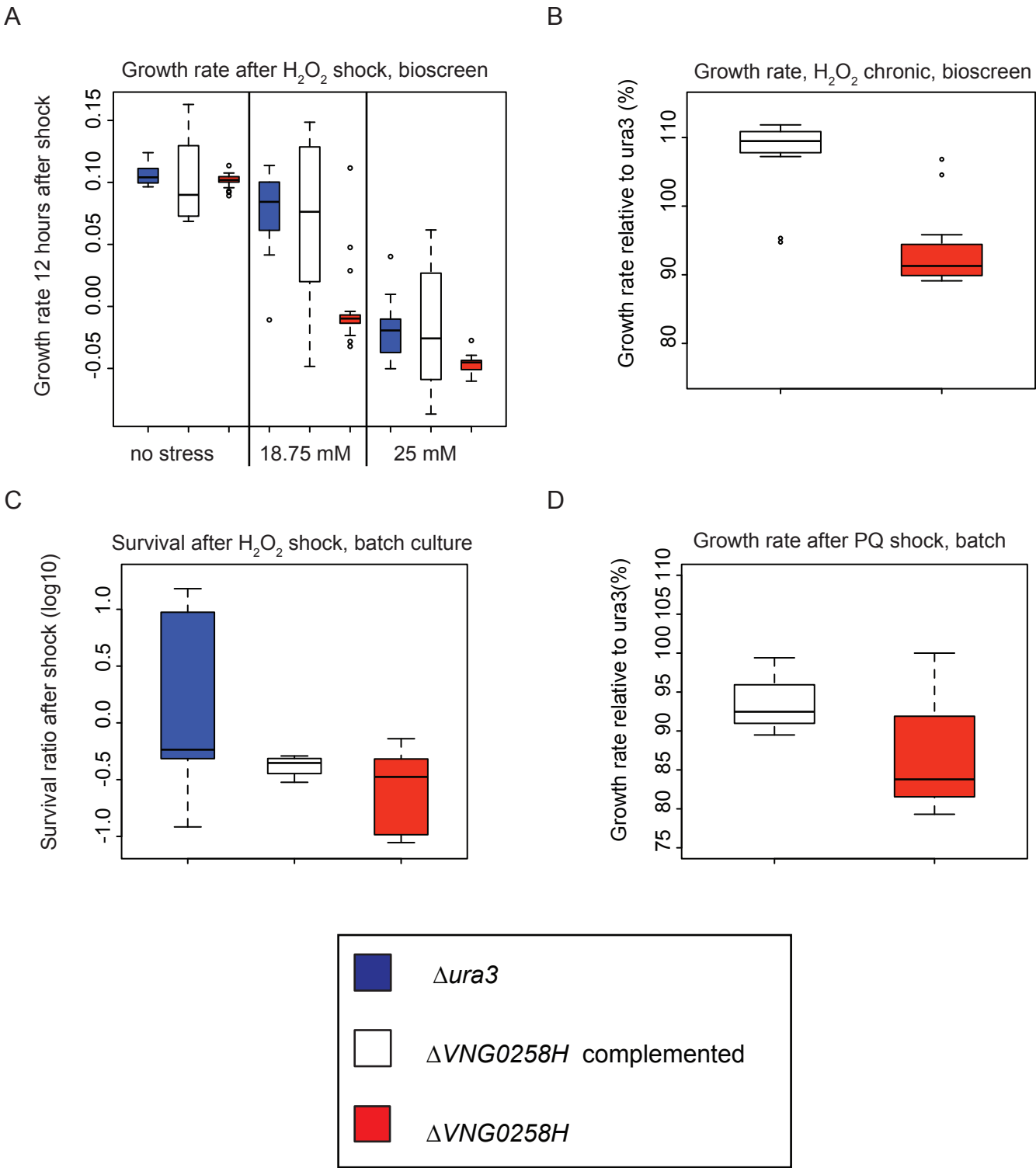

Supplement: Additional file 7 — Figure S2. A wild type copy of the VNG0258H gene supplied on a plasmid (pMTFcmyc vector, [1]) complements the ΔVNG0258H growth defects. (A) Box-whisker plots depicting growth rates of H. salinarum strains in the bioscreen C (Δura3 parent, ΔVNG0258H mutant, and ΔVNG0258H mutant complemented in trans) during the 12 hours following H2O2 shock (mid-logarithmic phase addition of H2O2). Horizontal lines within each box represent the median growth rate across 24 replicate trials (8 biological replicates, 3 technical replicates) for each strain in each condition. Boxes represent the interquartile range (IQR), and whiskers are minimum and maximum values within 1.5 times the IQR. Concentrations of H2O2 added are indicated on the X-axis, whereas the Y-axis quantifies growth rate. (B) Box-whisker plot depicting lag phase addition of H2O2 to Bioscreen cultures. Boxes, median lines, and whiskers are as in (A). Y-axis expresses the growth rate of the ΔVNG0258H or trans-complemented strains as a function of Δura3 growth rate. (C) Box-whisker plot depicting survival ratios 24 hours after mid-logarithmic phase addition of 25 mM H2O2 to batch cultures. (D) Box-whisker plot depicting growth rates following mid-logarithmic phase addition of PQ to batch cultures. Growth rates are expressed as a function of Δura3 parent strain growth. [file 1471-2164-13-351-S7.pdf]

Supplementary Figure 3.

A

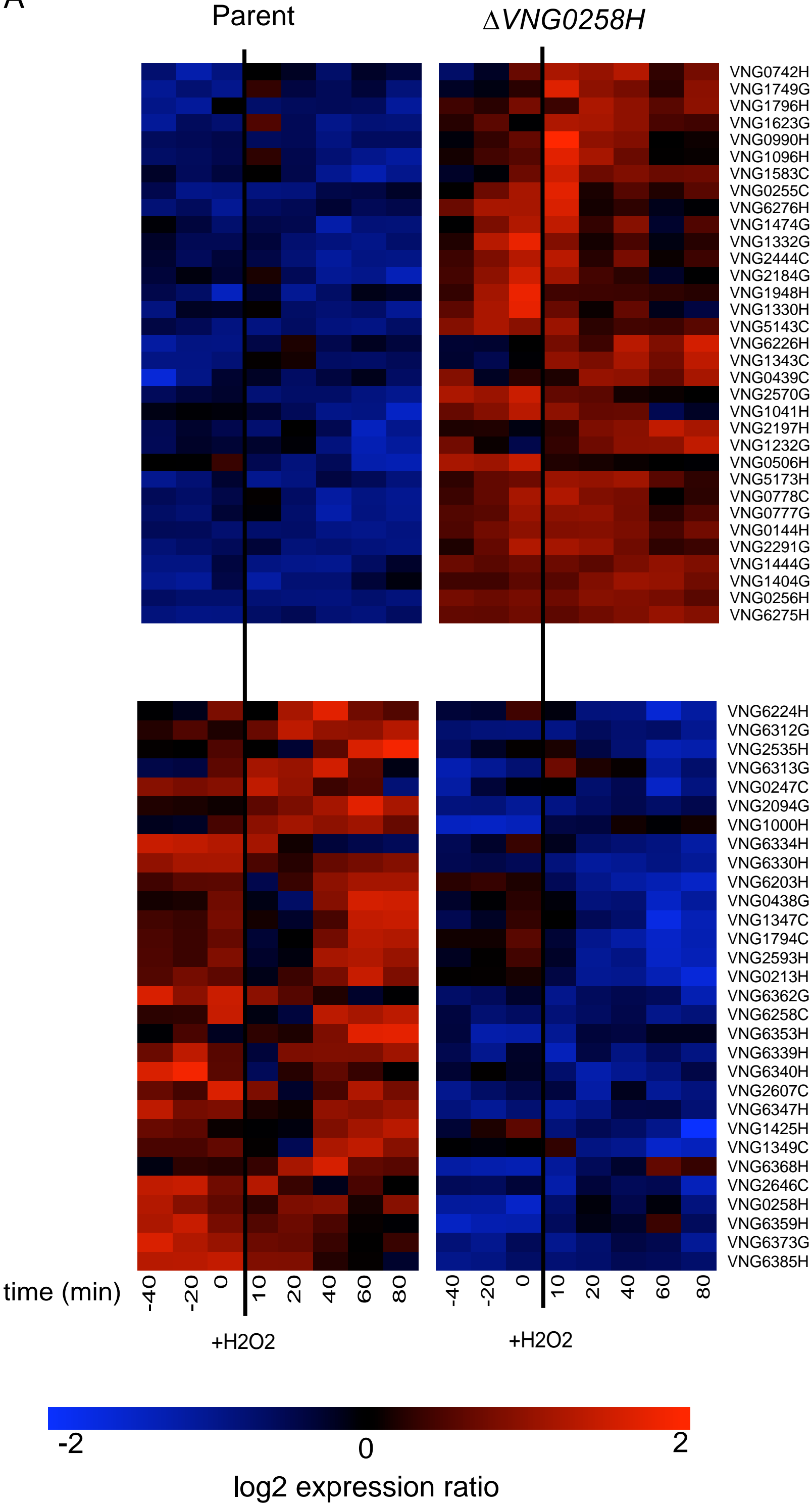

B

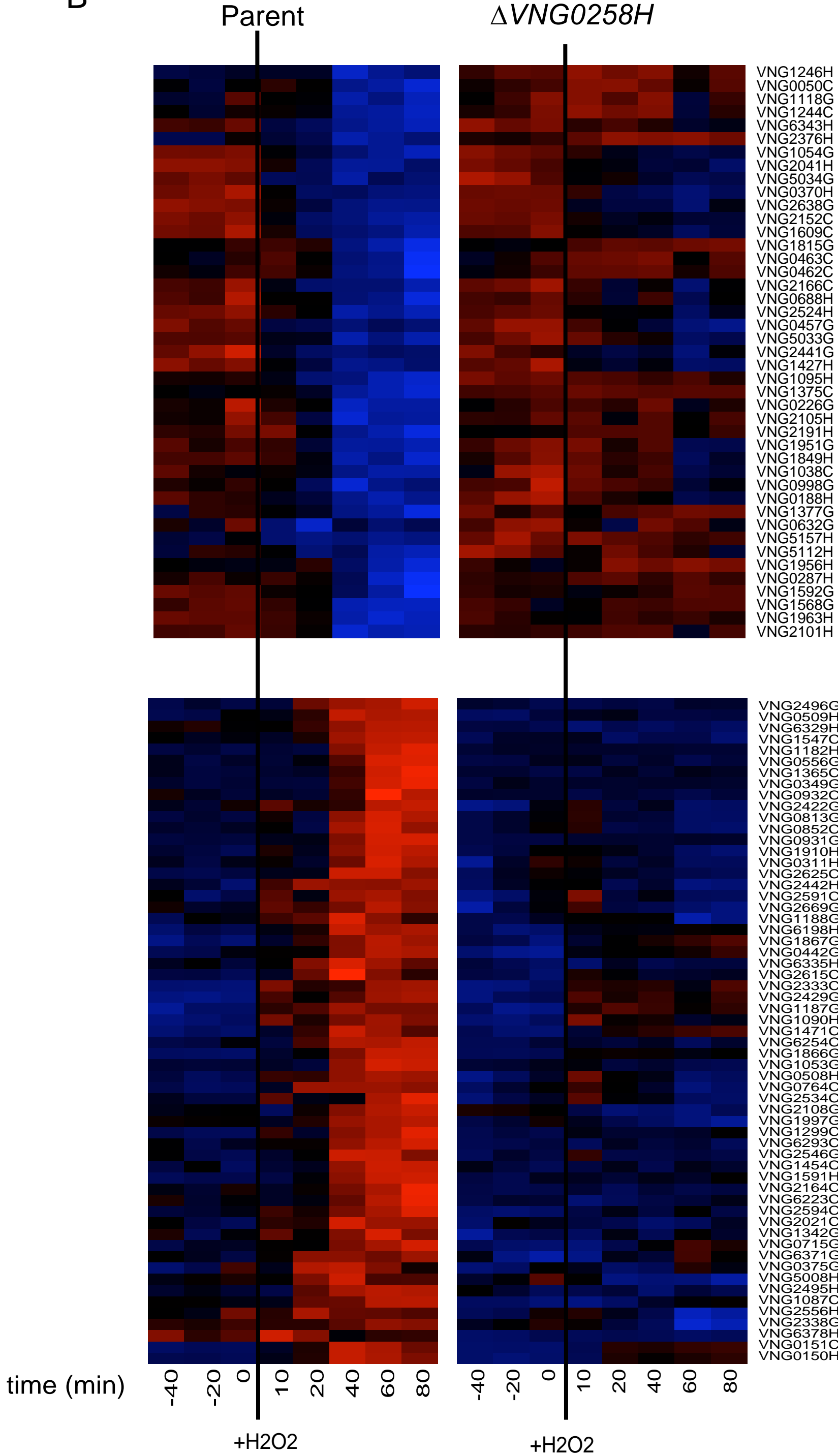

C

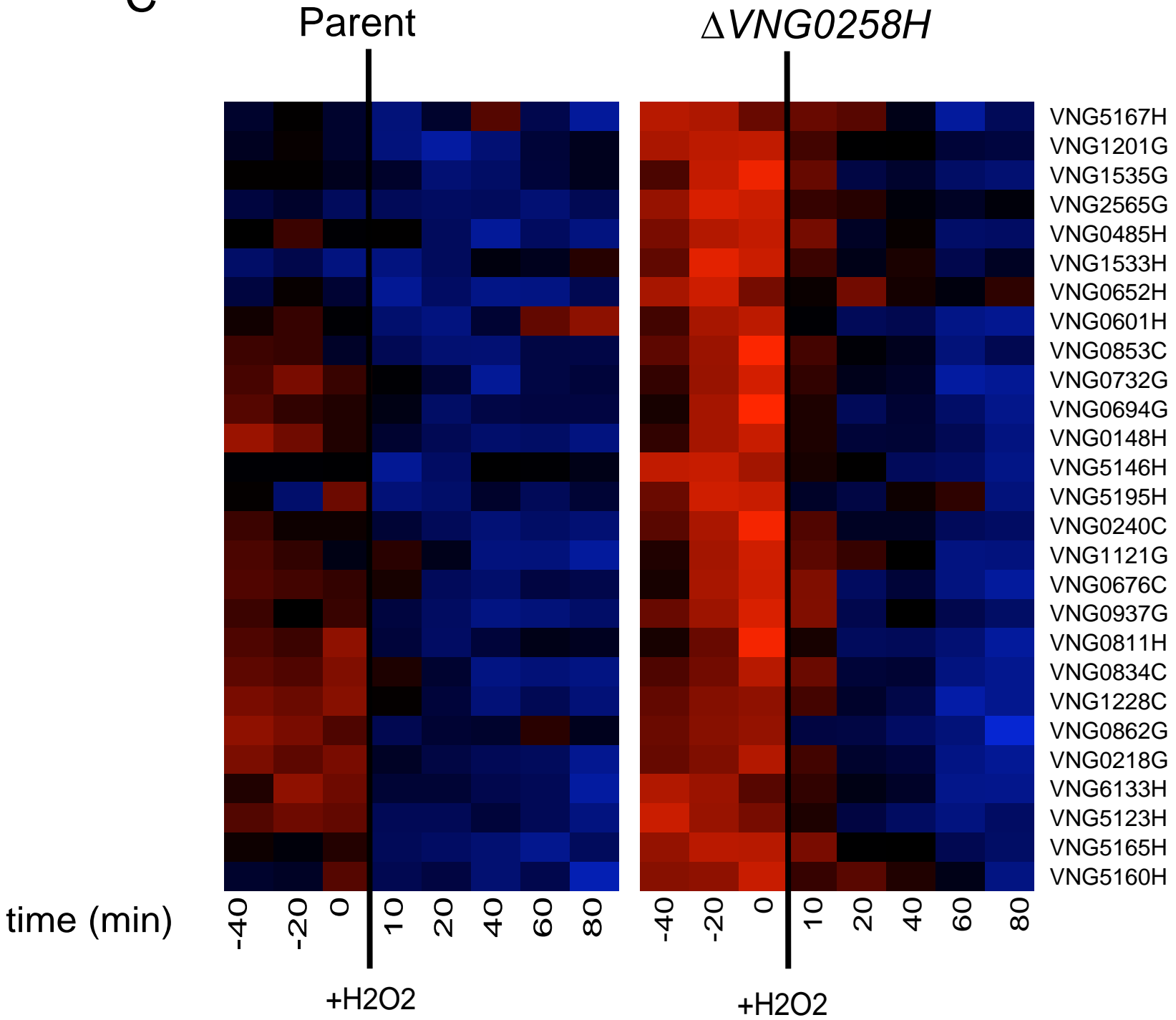

D

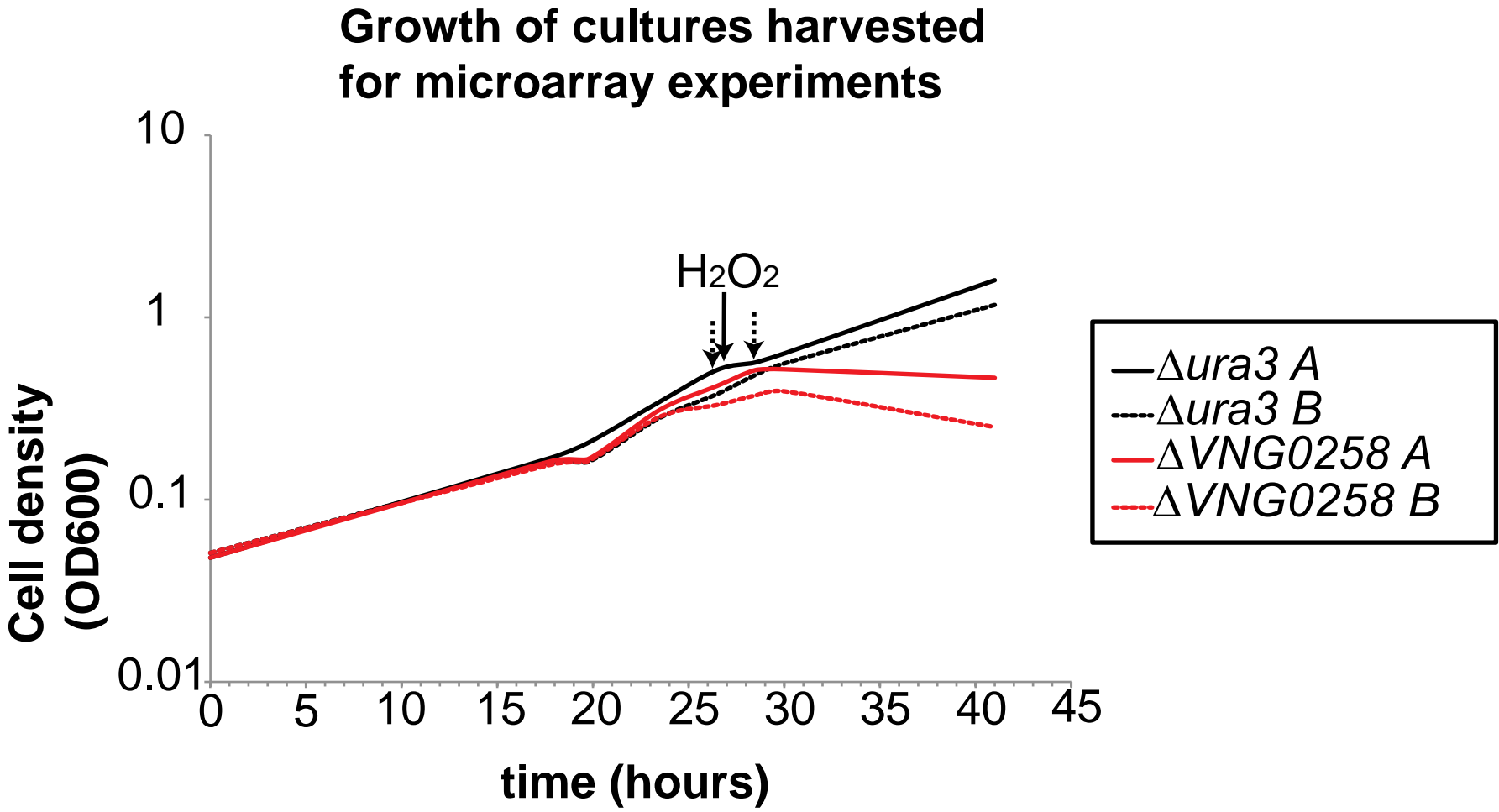

Supplement: Additional file 8 — Figure S3. Detailed heat map for each gene cluster from main text Figure 5 (H2O2 exposure). Data for those genes dependent on VNG0258H for appropriate expression are shown (i.e. main text Figures 5A-G). Gene names are listed on the right of each heat map. Detailed annotations and COG category memberships (main text Figure 7A) for each these genes are listed in Additional file 4: Table S2. In each heat map, red represents induction, whereas blue represents repression. VNG0258H-independent genes (Cluster 4, Figure 5H and J) are not included in the Figure for brevity and clarity, but expression data and annotations for these genes are included in Additional file 4: Table S2. (A) Cluster 1 includes genes that were differentially expressed in the ΔVNG0258H mutant vs Δura3 parent strain regardless of growth condition (main text Figures 5A-B). Cluster 1a (main text Figure 5A) depicts those 33 genes that are over-expressed in the ΔVNG0258H mutant (i.e. RosR is required to repress these genes). Cluster 1b (main text Figure 5B) depicts those 30 genes that are under-expressed in the ΔVNG0258H mutant (i.e. VNG0258H is required to activate these genes). (B) Cluster 2 includes genes that were differentially expressed in the ΔVNG0258H mutant vs Δura3 parent strain in the presence of H2O2 (main text Figures 5C-D). Cluster 2a (main text Figure 5C) contains those 43 genes that are over-expressed in the ΔVNG0258H mutant in response to H2O2 (i.e. VNG0258H is required to repress these genes in response to H2O2). Cluster 2b (main text Figure 5D) contains those genes that are under-expressed in the ΔVNG0258H mutant in response to H2O2 (i.e. VNG0258H is required to induce them). (C) Cluster 3 includes genes that were differentially expressed in the ΔVNG0258H mutant vs Δura3 parent strain in the absence of H2O2 (main text Figure 5E). (D) Growth of Δura3 parent and ΔVNG0258H cultures for gene expression microarray analysis. Black curves represent growth data for the two biological replicate [file 1471-2164-13-351-S8.pdf]

Supplementary Figure 4.

A

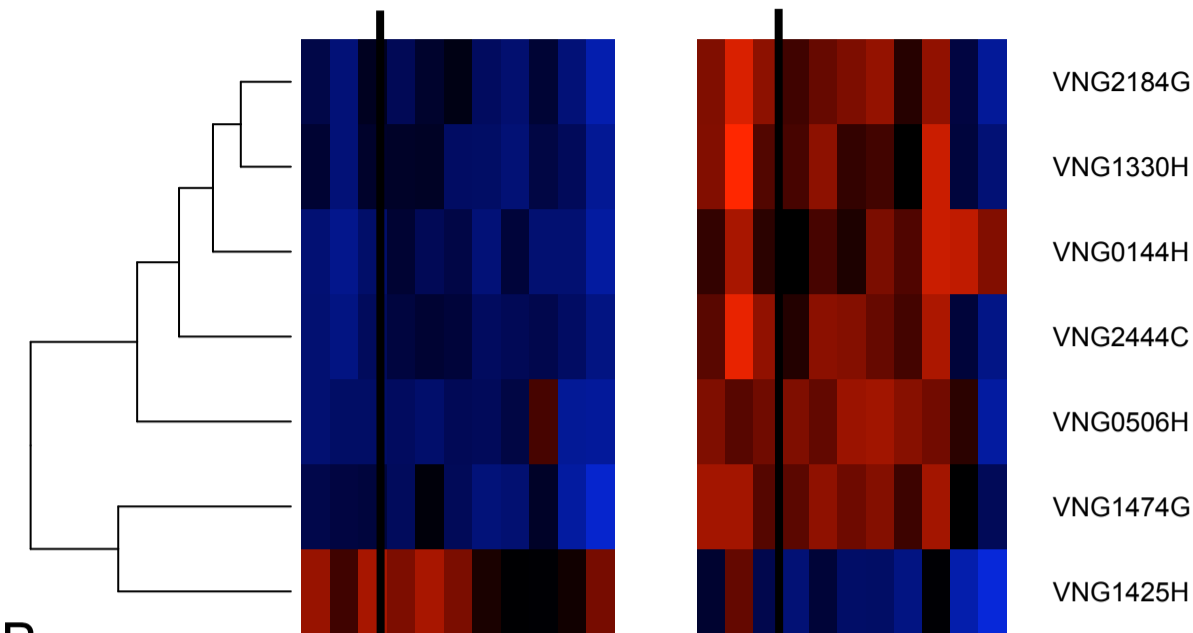

B

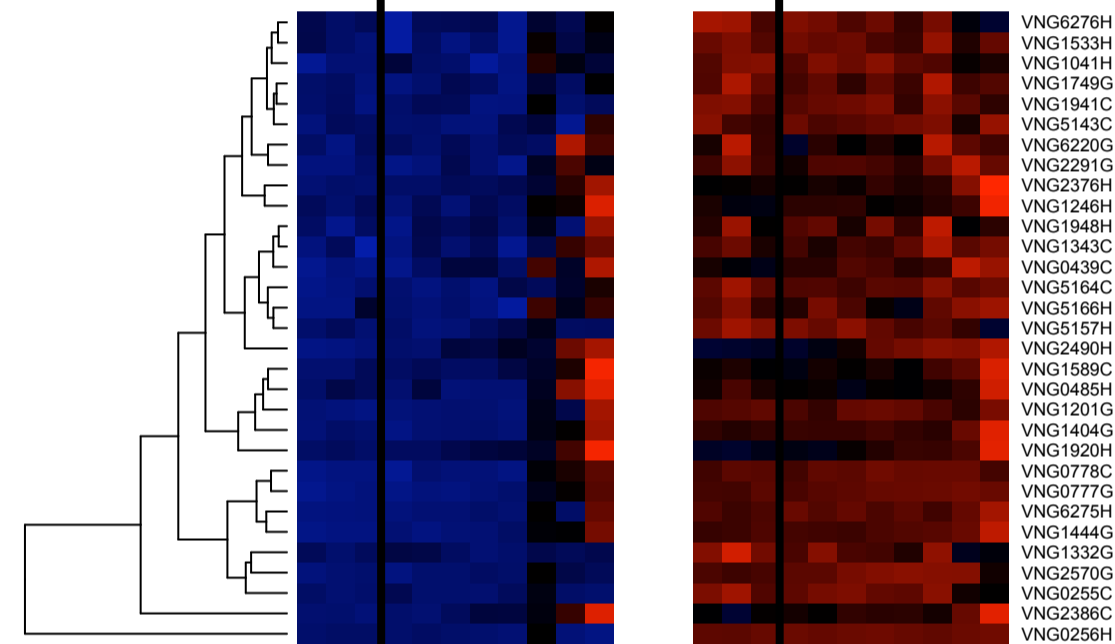

C

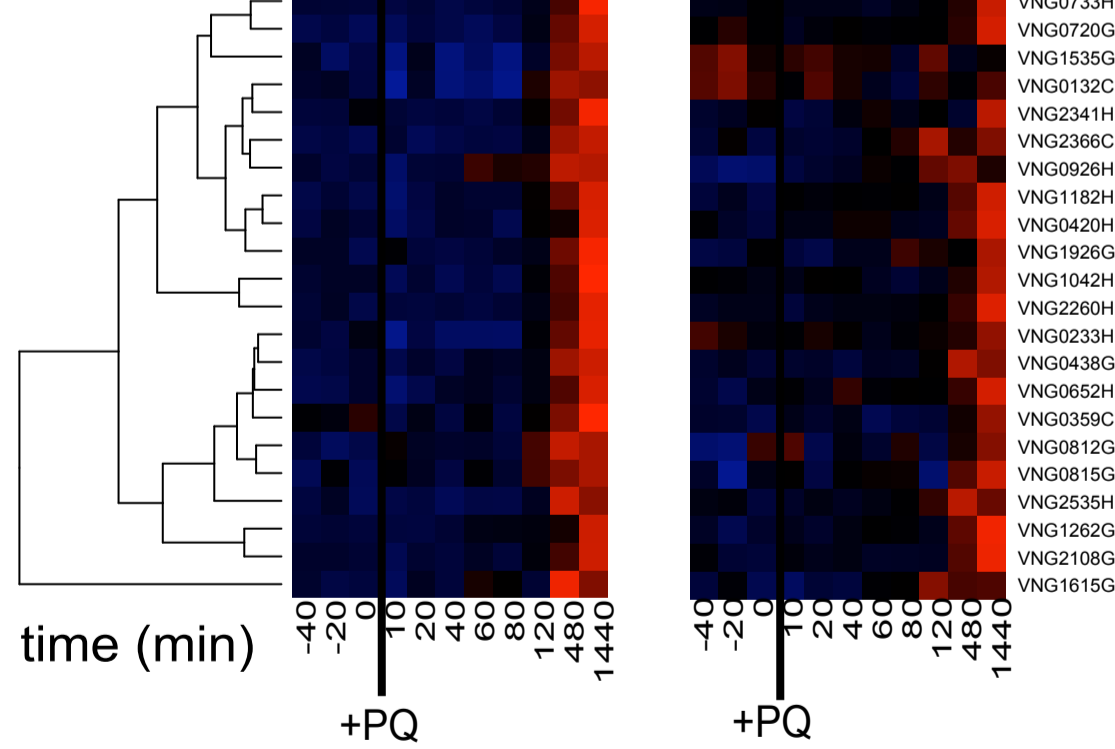

time (min)

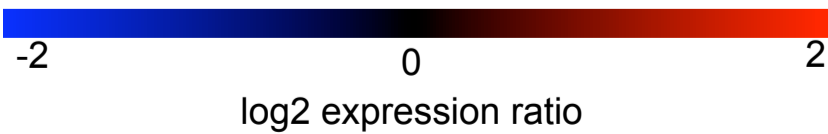

D

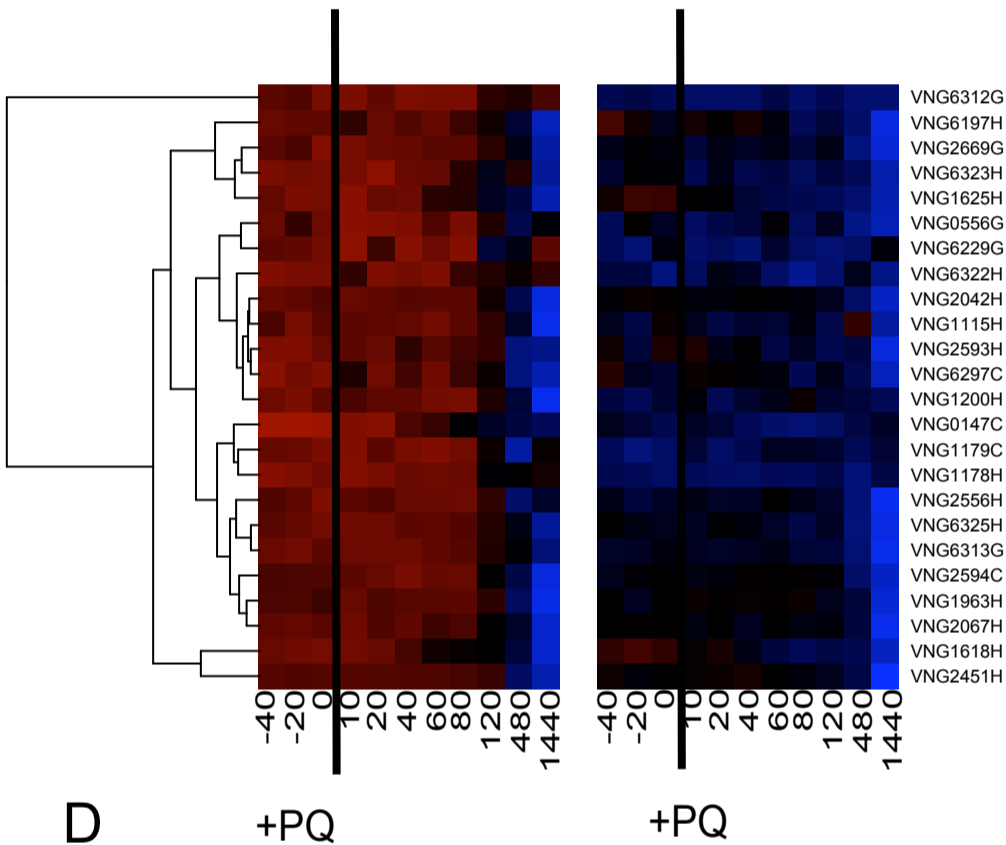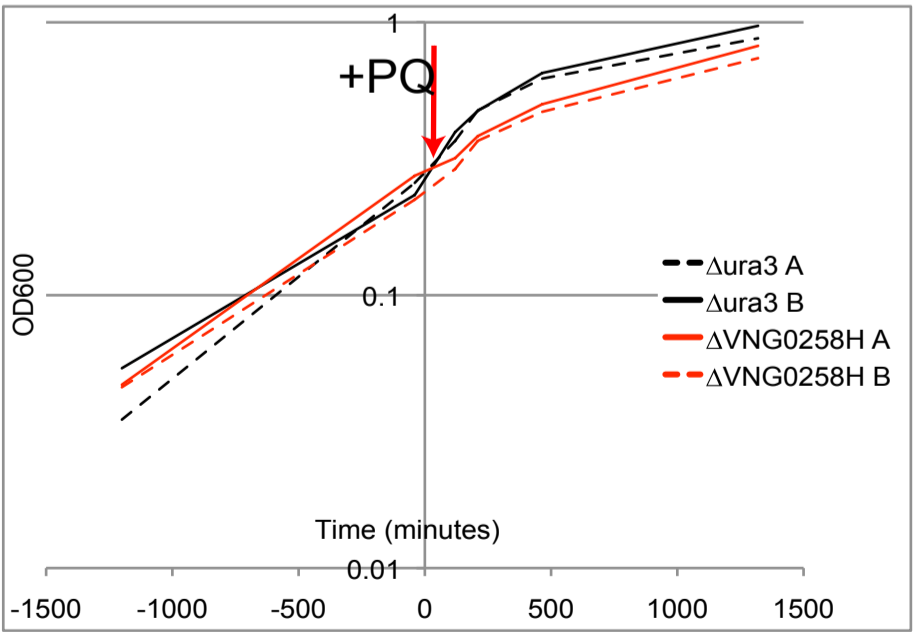

Supplement: Additional file 9 — Figure S4. Detailed heat map for each gene cluster from main text Figure 6. Data for those genes dependent on RosR for appropriate expression in response to PQ are shown (main text Figures 6A-C). Colors and labels are as in Additional file 8: Figure S3. (A) Heatmap for Cluster 1, genes differentially expressed in ΔrosR vs the Δura3 parent strain regardless of growth condition (main text Figure 6A). (B) Heatmap for Cluster 2, genes dependent upon RosR for differential expression in response to paraquat (PQ). Genes upregulated in the mutant are shown on the left (main text Figure 6B) and those downregulated are shown on the right (main text Figure 6C). (C) Genes differentially expressed in response to PQ that are independent of RosR. Upregulated genes are shown (main text Figure 6D). Downregulated genes (171 genes) are not shown for brevity, but are listed in Additional file 5: Table S3. (D) Growth data for cultures from which RNA was harvested for microarray studies. Red arrow indicates the time of PQ addition. [file 1471-2164-13-351-S9.pdf]

Supplementary Figure 5.

A

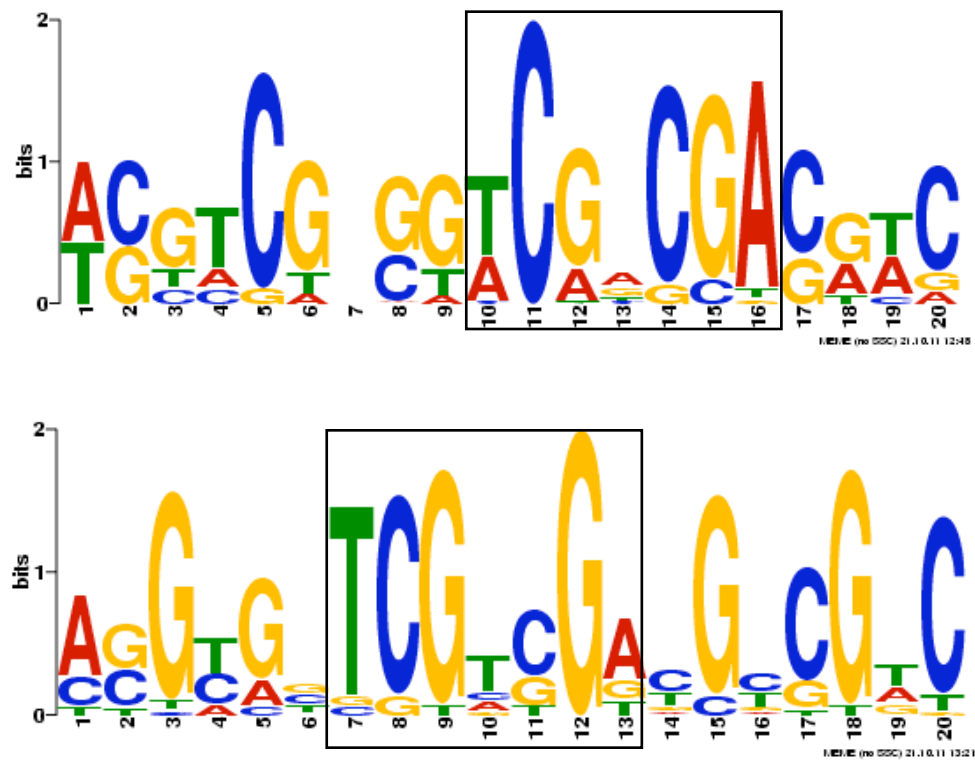

B

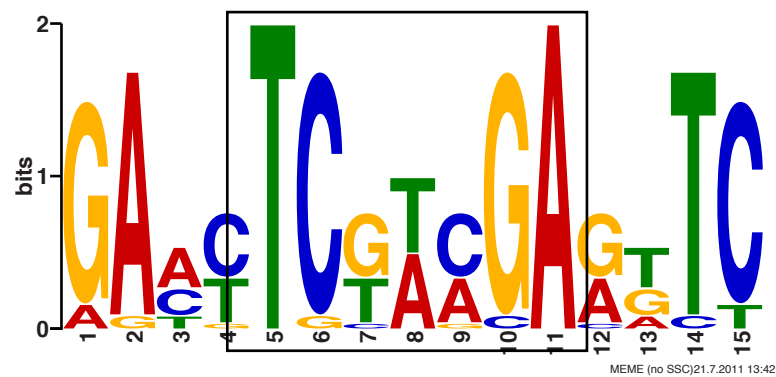

Supplement: Additional file 10 — Figure S5. Putative cis-regulatory sequences resulting from MEME analysis on (A) the 50 genes differentially expressed in common in the PQ and H2O2 gene expression datasets (main text Table 1), and (B) phylogenetic footprinting using sod2 promoter sequences from all halophilic archaea possessing a RosR homolog. Each sequence logo represents a different cis sequence prediction. The height of the letters in each nucleotide position represents the strength of the consensus between the input sequences. The putative TCG-N-CGA motif is boxed in each case. In (A), the top-scoring two motifs from MEME searches are shown. Top motif p-value is 7.0x10^-56^, and bottom motif p-value is 2.6x10^-42^. 43 of the 50 promoter query sequences contained each motif. In (B), only the top-scoring motif is shown. [file 1471-2164-13-351-S10.pdf]
